# Role of spinal sensorimotor circuits in triphasic muscle command: a simulation approach using goal exploration process

**DOI:** 10.3389/fncom.2026.1745836

**Published:** 2026-03-11

**Authors:** Daniel Cattaert, Matthieu Guemann, Florent Paclet, Luca Lemarchand, Bryce Chung, Pierre-Yves Oudeyer, Aymar de Rugy

**Affiliations:** 1 Institut de Neurosciences Cognitives et Intégratives d’Aquitaine, UMR 5287, CNRS, Université de Bordeaux, Bordeaux, France; 2 Neuroscience Institute, Georgia State University, Atlanta, GA, United States; 3 Inria Bordeaux Sud-Ouest, Talence, France

**Keywords:** goal exploration, motor control, sensorimotor circuits, spinal circuits, triphasic pattern

## Abstract

During rapid voluntary elbow movement on horizontal plane, a stereotyped triphasic pattern is typically observed in the electromyograms (EMGs) of antagonistic muscles acting at this joint. To explain the origin of such triphasic commands, two types of theories have been proposed. Peripheral theories consider that triphasic commands result from sensorimotor spinal networks, either through a combination of reflexes or through a spinal central pattern generator. Central theories consider that the triphasic command is elaborated in the brain. Although both theories were partially supported by physiological data, there is still no consensus about how exactly triphasic commands are elaborated. Moreover, capacities of simple spinal sensorimotor circuits to elaborate triphasic commands on their own have not been tested yet. In order to test this, we modelled arm musculoskeletal system operating in the absence of gravity, muscle activation dynamics, proprioceptive spindle and Golgi afferent activities and spinal sensorimotor circuits. Step commands were designed to modify the activity of spinal neurons and the strength of their synapses, either to prepare (SET) the network before movement onset, or to launch the movement (GO). Since these step commands do not contain any dynamics, changes in muscle activities responsible for arm movement rest entirely upon interactions between the spinal network and the musculoskeletal system. Critically, we selected step commands using a Goal Exploration Process inspired from baby babbling during development. In this task, the Goal Exploration Process proved very efficient at discovering step commands that enabled spinal circuits to handle a broad spectrum of functional behaviors, displayed in a behavioral space characterized by movement amplitude and maximal speed. All over the behavioral space, specific SET and GO commands elicited natural triphasic commands, thereby substantiating the inherent capacity of the spinal network in generating them.

## 1 Introduction

During rapid voluntary limb movement about a single joint, a stereotyped triphasic pattern is observed in the electromyograms (EMGs) of antagonistic muscles commanding this joint (Hallett et al., 1975). Note that, in the Hallett et al. study, elbow flexion was produced in horizontal plane (i.e., arm was abducted approximately 90° at the shoulder), a movement in which gravity plays no role. Prior to limb displacement, the agonist muscle is briefly active (AG1). After the limb begins to move, a burst of muscle activity occurs in the antagonist muscle (ANT) while the agonist is silenced, and then a second burst is observed in the agonist (AG2). The precise organization of this triphasic pattern ensures the smoothness of the movement.

What is the origin of this triphasic command? Two possibilities have been proposed; either a spinal origin via sensorimotor circuits (Barnett and Harding, 1955; Terzuolo et al., 1974; Ghez and Martin, 1982) or a central origin elaborated in brain networks and transmitted to the spinal cord via descending commands. This debate also contrasted the *λ*-model (Feldman, 1986, 2019) with the central origin theory (Corcos et al., 1989; Gottlieb et al., 1989). The λ-model states that active movements may result from changes in reflex parameters such as the threshold and gain by control signals conveyed by descending systems. Specifically, in the λ-model, it is suggested that fast changes in the limb position are produced by a rapid monotonic change in the reflex threshold. The central hypothesis was supported by observations on humans with severe, though not complete, sensory neuropathy, who could still produce triphasic patterns (Hallett et al., 1975; Rothwell et al., 1982). Although these studies highlight the capacities of the motor cortex and other higher centers that can learn how to control limb movements after partial or total deafferentation, the deep reorganization of motor commands following such deafferentation prevents firm conclusion about how triphasic commands are produced in healthy subjects. Other studies used reversible transient deafferentation using ischemic process of the muscles controlling the joint and their afferent nerves Ia and Ib, the diameter of which are more sensitive to ischemia than axons of alpha motoneurons (Sanes and Jennings, 1984). During such reversible ischemia, the triphasic pattern persists, although passive movements fail to elicit stretch reflexes.

Other experiments indicate that triphasic motor command is also present in the primary motor cortex (M1), and that neural activities in M1 are correlated with output kinematics and kinetics during isometric-force and arm-reaching tasks (Sergio et al., 2005). Physiological model describing the transformation from the neural activity in M1, through the muscle control signal, into joint torques and down to endpoint forces and movements (Trainin et al., 2007), show that optimized solutions for M1 activities display triphasic changes in temporal pattern and instantaneous directionality similar to that found in the EMG (Trainin et al., 2007).

In opposition with the arguments in favor of a central origin of triphasic commands, it was shown that procedures that modify discharge of muscle spindle afferents during movement alter the duration and amplitude of these bursts (Hallett and Marsden, 1979; Brown and Cooke, 1981). Moreover, the involvement of spindle afferent signals in the AG1 burst was demonstrated in experiments showing that the onset time of AG1, even in the fastest movements, depends on proprioceptive feedback in a manner consistent with the stretch reflex (Adamovich et al., 1997) and incompatible with long-delay cortico-spinal loops. In addition, simulation studies concerning the central origin of triphasic commands, although aiming toward physiological models, often lack spinal sensorimotor loops (Trainin et al., 2007). Therefore, if we cannot doubt that M1 neurons and populations of neurons present activities correlated with kinetic, kinematic and dynamic cues of the performed movements, there is still questions about the possible role of spinal sensorimotor circuits in the elaboration of triphasic commands. Notably, since both efference copy from spinal interneurons and afferent feedback from proprioceptors project onto sensorimotor cortical areas, they constitute a potential origin for triphasic patterns observed in cortical areas. Consequently, if the first EMG activity (AG1) can be attributed to a central command from the brain (at least for its onset), there is still debate about ANT and AG2.

The aim of this paper was to explore the capacities of the spinal sensorimotor circuits in shaping triphasic commands, using a modeling approach based on the neuromechanical environment AnimatLab (https://www.animatlab.com/). We modelled the movement of the forearm around the elbow joint, involving two muscles (biceps and triceps). Movements consisted in elbow flexion starting from a fully extended position. Given our aim, we refrained from using descending commands containing any dynamic or timing information as this would defeat the very purpose of testing the capacity of spinal network circuits to produce a triphasic pattern on their own. Indeed, how could this capacity be demonstrated should critical information about the triphasic pattern be already contained in the descending commands sent to spinal networks? Thus, rather than positing a cortical origin for the dynamics underlying the triphasic command, we explored conditions under which sensorimotor circuits could independently generate the complex muscle activity patterns observed during rapid arm flexion in humans. Several modelling studies of spinal cord circuits controlling elbow movements, and receiving simple descending step commands have been published (Raphael et al., 2010; Parziale et al., 2020). These two modeling studies share many common features (spinal sensorimotor circuits, target of descending step commands… etc.). But, whereas Raphael et al. (2010) in their spinal like regulator (SLR) model use SET commands to prepare spinal sensorimotor networks without eliciting any movements and GO commands delivered at a single later time, Parziale et al. (2020) use step commands acting at three different time points. For a review of spinal cord models see Parziale and Marcelli (2024). In order to fulfill our criteria of absence of dynamics in the descending commands, our approach is inspired from the work of Raphael et al. (2010). In short, spinal interneurons and synapses of sensorimotor circuits were controlled by simple SET and GO step commands. The SET commands shape the sensorimotor circuits (synaptic gains, level of activity of neurons) without producing any movement, and the GO commands trigger and elicit the movement. Since these signals lacked any dynamic content, the spinal sensorimotor circuit was solely responsible for shaping the dynamics of the commands, particularly in producing the triphasic commands.

We also wanted the neuromechanical system to self-organize its commands as a function of the produced movements, as would do a child exploring the commands of its own arm. Recent advances in developmental machine learning (Baranes and Oudeyer, 2013; Forestier et al., 2017) have shown that a novel form of exploration algorithms, called *Goal Exploration Processes* (GEP), can be used to automatically define target behaviors and efficiently discover a map of reachable behaviors, thereby solving problems of unreachable behaviors and local minima encountered by alternative, state-of-the-art optimization methods (Oudeyer and Kaplan, 2007; Forestier et al., 2017; Colas et al., 2018); (for related methods using diversity search to solve problems with local minima or sparse rewards, see also Lehman and Stanley, 2011; Mouret and Doncieux, 2012; Pathak et al., 2017; Eysenbach et al., 2019). Moreover, self-conducted exploration of high-dimensional, continuous parameter systems have the advantage to be sample efficient, i.e., they enable the discovery of diverse behavioral domains with a limited number of experiments. So far, such algorithms have been used to discover diverse behaviors in robotics (Forestier et al., 2017; Péré et al., 2018) as well as in complex systems such as cellular automata, morphogenetic models (Reinke et al., 2019; Etcheverry et al., 2020) and chemical systems (Grizou et al., 2020), implementing a form of discovery assistant that can leverage interactively guidance of users (Etcheverry et al., 2020). Furthermore, GEP is not only an exceptional tool for exploring behavioral domains, but this type of exploration, where an agent defines its own goals, has recently been recognized as a curiosity-driven learning mechanism. This approach is used in artificial intelligence and machine learning, similar to how it occurs in animals and humans (Gottlieb and Oudeyer, 2018).

Finding new sets of parameters and behaviors from previously achieved behaviors has already been successively used to generate intermediate movement using interpolation (Tsianos et al., 2014). However, due to the large redundancy of the neural circuits, interpolation fails if previous behaviors relate to different strategies (Tsianos et al., 2014). To get a systematic exploration of the capacities of the neuromechanical model, we used a simple GEP protocol inspired from Forestier et al. (2017). These algorithms use two work spaces: (i) one for the behaviors: here we used a two dimensions space (movement amplitude and movement speed), and (ii) one for the parameters that determine the motor commands from the brain: here we used a series of step commands that modify synaptic strength and neuron activity in spinal cord circuits either in the preparatory phase (SET) or to launch the movement. The GEP finds new valid movements by repeating 4 stages: (1) Choose a goal (a goal is a targeted behavior); (2) Find the closest already known valid movement to this goal; (3) Modify slightly these parameters in a random way; (4) Run the model with the new parameters; (5) Observe the result and if acceptable, store the valid movement and parameters in the database.

Several modelling approaches have proposed that the CNS employs a minimum jerk strategy when planning any given movement (Flash and Hogan, 1985). This proposal was refined later (Nakano et al., 1999) showing that minimum angle jerk predicts the actual arm trajectory curvature better than the minimum jerk model. Note that even if not explicitly specified, minimum jerk profiles are obtained in feedforward control-based models such as the minimum noise model (Haruno and Wolpert, 2005). Therefore, we used it here. Once valid movements were found, the GEP searched new ones by modifying parameters of preexisting valid movements. Because the policy was random, we called this process rGEP. Here, rGEP proved efficient at discovering step commands that enabled spinal circuits to handle a broad spectrum of functional behaviors while eliciting natural triphasic commands over the whole range of the behavioral domain.

## 2 Methods

### 2.1 Software’s used in simulations

During this work, we have used several models of sensorimotor systems controlling the same musculoskeletal apparatus. All models were developed with the AnimatLab software (https://animatlab.com) (Cofer et al., 2010), a neuromechanical environment based on user-friendly graphic interface that can be downloaded from https://www.animatlab.com. Execution of AnimatLab models can be controlled by Python scripts, which were used to command execution of model movements by step command targeting spinal neurons and synapses (see below). All scripts used in the present work are written in Python3.8 language. For a quick overview of GEP principles, see pseudo script in Supplementary material. Python scripts for GEP and all simulations included in this study can be downloaded from GitHub, with an installation procedure for all software package involved:

(https://github.com/Cattaert/rGEP/tree/main).

### 2.2 Musculoskeletal system

**Figure 1:**
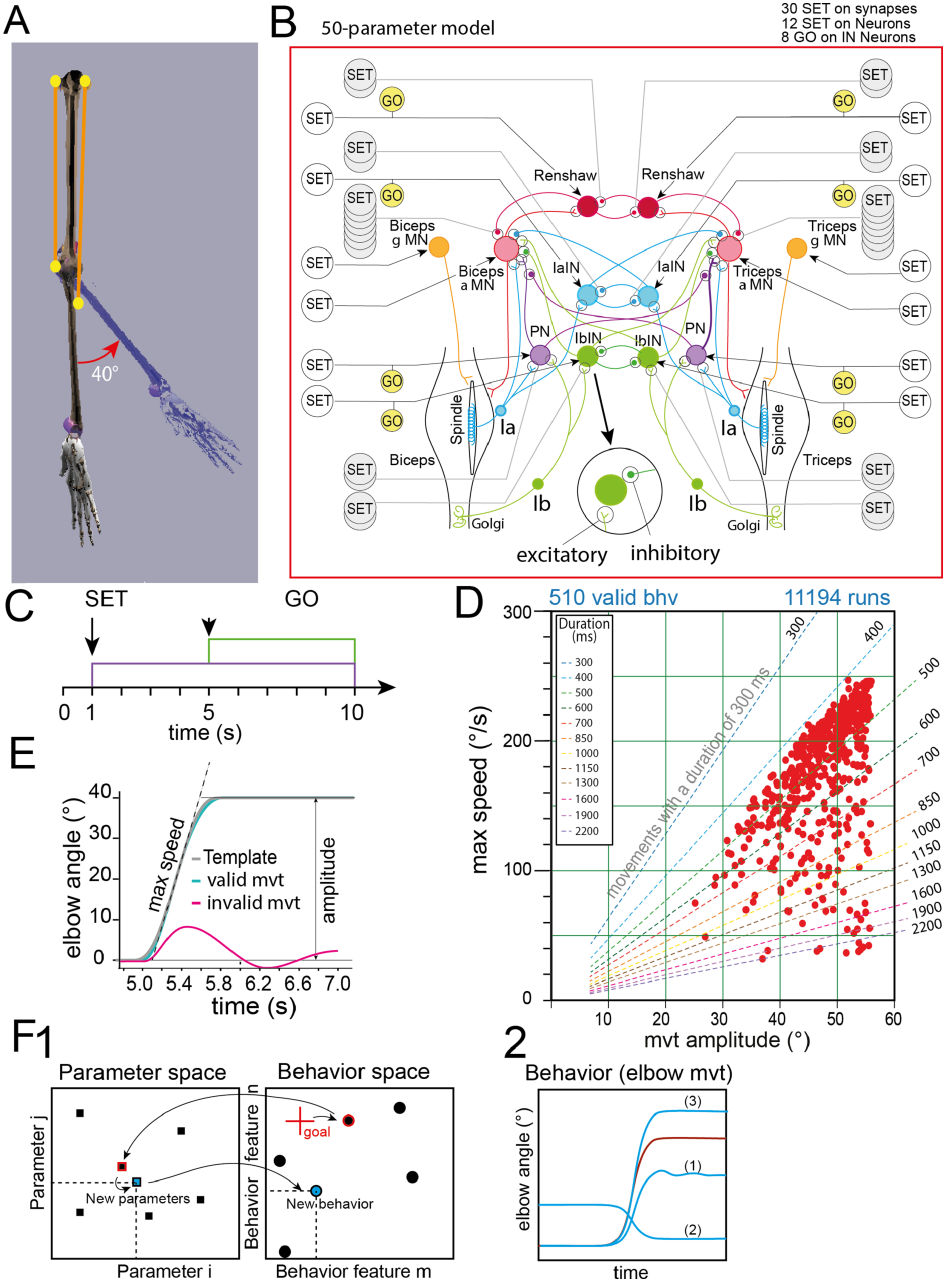
Neuromechanical model and GEP principles. **(A)** Musculoskeletal system. **(B)** Sensorimotor system comprising αMNs, γMNs, propriospinal interneurons, and Ia afferents from muscle spindles, with SET ad GO commands. In this study, simplified sensorimotor networks have been associated to the same musculo-skeletal system (see Figures 4–8). The inset indicates excitatory (∨) and inhibitory (•) synapse representation. **(C)** Timing of SET and GO commands used to control the sensorimotor network. The SET commands adjust all neuron activity levels at 1 s to bring the network in a state ready to trigger the movement (without producing the movement by itself). The GO commands launch the movement. SET and GO commands are the parameters of parameter space. **(D)** Example of behavior domain obtained with rGEP algorithm after 39,160 iterations. Valid movements are indicated by red dots, and corresponding movement durations are indicated by dashed lines. The behavior domain is the collection of valid movements represented in the behavior space defined by amplitude (abscissa) and maximal speed (ordinate) of elbow movements. **(E)** Valid movements (green curve) are movements fitting a minimum jerk profile (gray curve) [i.e., mean square error (MSE) < 1 - see Methods]. An example of rejected movement is presented (red curve). **(F)** The parameter space is a *n*-dimension space (*n* variables of each parameter set), defined as the ensemble of parameter sets associated with valid movements. In this parameter space, we keep all parameter sets associated with valid movements. We present here a schematic diagram of the GEP exploration algorithm. **(F1)** Process starts from an existing *parameter space* (black filled squares in *parameter space* – for simplicity reduced to two parameters), which is associated with a *behavior space* (black filled circles in *behavior space*), a new *goal* is given in the periphery of the known behavioral space (red cross). The algorithm searches for the closest behavior (closest neighbor), it will retrieve the parameters associated with this behavior and replay its parameters including additional noise (“*New parameters”*). This new set of parameters generates a “*New behavior”* in the space of behaviors that is kept or not, depending on its *Cost* value. **(F2)** The movement that was closest to the goal (red circle in behavior space, and red movement trace in **F2**) is a valid movement. However, when its parameters are changed randomly different issues are possible: (i) the obtained new movement is invalid, either because there are oscillations (1) or because it is performed in the wrong direction (2); (ii) the new movement is valid and may be close or far from its parent.

The musculoskeletal model consisted of an upper limb involving an elbow actuated by two antagonistic muscles (biceps and triceps) (Figure 1A). This single musculoskeletal model was used in all simulations, with various spinal sensorimotor circuits and step commands controlling their state (see below). Information about this musculoskeletal model is given in Table 1. Given that bones are the only elements on which we can play in the AnimatLab model, we used a bone density of 8,000 kg/m3 in order to obtain a correct total mass for each segment (with its bones and soft tissues). The anthropometric tables (Clauser et al., 1969) give the mass for the adult human upper arm (1.36–2.30 kg), forearm (0.85–1.38 kg) and hand (0.33–0.54 kg). For example, using a radius-cubitus density of 8,000 kg/m3, resulted in a simulated forearm mass of 0.802 kg, which is acceptable for our purpose.

**Table 1:** Musculoskeletal model data.

Segments	Length (mm)	density	mass
Humerus	337.0911395	8,000 Kg/m3	1.164 Kg
Forearm	283.1808226	8,000 Kg/m3	0.802 Kg
Hand	193.21	8,000 Kg/m3	0.966 Kg

Joints (coordinates world positions)	Z_coord (deepness)	Y_coord (up-down)	X_coord (left–right)
Elbow	−4.586	−144.65	−5.372
Wrist	14.883	−427.07	1.789
Muscle insertions (world positions)			
Biceps origin	0	150.69	26.509
Biceps insertion	−0.578	−188.28	24.146
Triceps origin	0	151.13	−21.51
Triceps insertion	0.934	−103.64	−16.507
Biceps insertion/Forearm elbow axis	52.82947367		
Triceps insertion/forearm elbow axis	42.85182289		

Coordinates and dimensions are given in mm. The origin of coordinates (0,0,0) is the center of the Humerus, that is in vertical position at rest (see Figure 1A).

All movements were produced in the absence of gravity in order to avoid natural slowdown of movement speed as the forearm is flexed. Note that this configuration meets the criteria used in Hallett et al. (1975). Muscles used in AnimatLab are based on the Hill muscle model (Scovil and Ronsky, 2006). The parameters of the Hill model for biceps and triceps are given in Table 2 with length/tension and stimulus/tension parameters. These parameters were chosen to match physiological data on the percentage of tension generated by the human biceps and triceps throughout the entire elbow flexion movement (Murray et al., 2000). These parameters were set in order to prevent force from being elicited when muscle is at rest on all muscle lengths displayed from total elbow extension (0°), to full elbow flexion (120°).

**Table 2:** Hill muscle parameters used in models for biceps and triceps.

Parameters		Biceps	Triceps
B		1 Ns/m	1 Ns/m
Kpe		100 N/m	100 N/m
Kse		1,000 N/m	1,000 N/m
Length		33.898 cm	27.25 cm
Max-tension		400 N	300 N
Length-tension	Lwidth	14 cm	35 cm
	Resting length	38.5 cm	38.5 cm
	Range of length	25–34 cm (120–0°)	27.5–34.5 cm (0–120°)
	Range of tension%	10–90%	90–100%
Stimulus-tension	Amplitude	400 N	300 N
	Steepness	100	100
	X offset	−20 mV	−30 mV
	Y offset	−4.5 N	−4 N

### 2.3 Presentation of the sensorimotor networks and their control by step commands

Non-spiking (NS) neurons were used to simulate neurons in spinal sensorimotor networks. NS neurons allow a continuous synaptic activation that depends only on presynaptic threshold potential, presynaptic saturation potential, equilibrium potential for post-synaptic effect and synaptic gain (maximal synaptic conductance). The change in postsynaptic conductance increases from 0 (at presynaptic threshold potential) to the maximal synaptic conductance (for presynaptic saturating potential and over). Between these presynaptic threshold and saturation potentials, the postsynaptic conductance increases linearly. Such neurons present the advantage to avoid discontinuity of spikes and spiking synaptic transmission. This is specially critic in small neuron networks, when the level of activity is low. Using NS neurons can therefore be considered as representing the average activity of populations of spiking neurons instead of single neurons (Strohmer et al., 2021).

In this study, we have used several sensorimotor spinal networks, all derived from the “complete” model (Figure 1B). The complete spinal network (50 parameters model, Figure 1B) received proprioceptive feedback from muscle spindles (Ia) and Golgi Tendon Organ (GTO). The associated sensorimotor circuits were inspired from Raphael et al. (2010), and contained 12 neurons [2 alpha motoneurons (MNs), 2 gamma MNs, 2 propriospinal interneurons (INs), 2 Ia INs, 2 Ib INs, and 2 Renshaw INs], and 30 synapses.

The specific aim here was to generate minimum jerk movements (i.e., fitting to this movement shape was in the cost function – see below). We used the same principles for step commands as Raphael et al. (2010). Step commands were represented as step functions starting at *t* = 1 s (SET command) and at *t* = 5 s (GO command) (Figure 1C). Like in the model of Raphael et al. (2010), SET and GO commands were maintained up to the end of the simulation (*t* = 10s). The SET commands were responsible for the state of readiness of the network. This first stimulation should activate the network without releasing any movement (Prut and Fetz, 1999; Eliasmith and Anderson, 2003). The cost function (see Methods 6) naturally ensured that SET commands did not elicit any movement. More specifically when the SET and GO commands are executed, the cost function is used to verify that no movement is produced by the SET command, simply by measuring the evolution of elbow angle during the trial. In the cost function defined in Equation 1, a movement penalty is included as follows:


$$\sum_{t=2}^{t=10}{\left(T_{\text{desired}}(t)-T_{\text{actual}}(t)\right)}^{2}$$

As the desired elbow angle is maintained at 0° from *t* = 2 s to *t* = 5 s, any change of elbow angle during the preparatory phase would result in a high penalty preventing the movement from being selected.

Please note that the precise timing between the SET and the GO commands is not important, providing that their order of appearance is respected (i.e., SET before GO), and that the time in between is sufficient for the state of readiness to be reached before movement launching. This was achieved using 4 s between the SET (*t* = 1 s) and the GO (*t* = 5 s) commands.

A part of the SET commands is directed toward neurons (interneurons, alpha MNs, and gamma MNs) to regulate their activity levels (Prut and Fetz, 1999; Eliasmith and Anderson, 2003), while another part of the SET commands controls the strength of spinal synapses (Burke et al., 1992b; Rudomin and Schmidt, 1999; Chen et al., 2002; Carroll et al., 2005). Among the neuronal target of SET commands, propriospinal interneurons (PN in Figure 1B) seem to play an important role since they connect most of the arm MNs (Alstermark et al., 1999) via excitatory and inhibitory pathways in human (Iglesias et al., 2007). PN interneurons receive excitatory projections from pyramidal neurons (Isa et al., 2006), and their excitability is controlled during the preparatory phase in human (Baldissera and Pierrot-Deseilligny, 1989; Burke et al., 1992a). Group Ia reciprocal inhibitory interneurons involved in limb flexion/extension receive synaptic input from multiple descending systems, including vestibulospinal and corticospinal pathways (Jankowska et al., 1976; Kudina et al., 1993), while group Ib interneurons receive prominent input from corticospinal and rubrospinal tracts (Harrison and Jankowska, 1985). The excitation of these inhibitory interneurons is modulated during the preparatory phase of the movement.

The GO commands were responsible for movement initiation. In human, spinal interneurons, notably PN receive descending input at the initiation of movement (Burke et al., 1992a). In our model we used the same supraspinal control for the GO commands as in (Raphael et al., 2010). Briefly, GO command modulated the level of activity of propriospinal interneurons (PN), 1a interneurons (IaINs), 1b interneurons (IbINs) and Renshaw INs (Renshaw) (see Figure 1B). Note that at *t* = 5 s, the transient changes induced by the SET command had stabilized before the GO command was issued. In summary, GO commands targeted the 8 INs, SET commands targeted the 12 neurons and adjusted the 30 synapses’ gains.

### 2.4 Brief overview of rGEP

To assess behavior capabilities associated with a given neuromechanical model [i.e., the variety of valid movements that can be produced (Figure 1D)], and meet some criteria like minimum jerk (Figure 1E - see below for the cost function), we have developed a rGEP algorithm that uses two spaces (the parameter space and the related behavior space). The behavior space is a two dimensions space (amplitude and the maximal speed of elbow movements). In this space, the collection of all found valid movements is referred to as behavior domain. At each iteration, goals (i.e., movements of given amplitude and speed) are self-produced by the algorithm. Goals are disposed either on the periphery of (extend strategy) or inside (fill strategy) the known behavior domain. Generally, the new valid behaviors are in the vicinity of the targets, with a variation due to the random process. They may be inside the domain of outside the domain. In the extend strategy, targets are chosen on the periphery of the behavior domain explored at the beginning of a given trial, thereby maximizing the probability to find new behaviors that will extend the behavior domain due to the random changes made to the source parameters associated with these targets. The fill strategy aims at filling the empty spaces (holes) within the behavior domain. In this strategy, the target movements are chosen around the holes. The new valid behaviors will therefore maximize the probability to find new valid movements in these holes. Formal expression of the extend and fill strategies are provided as pseudocodes in the Supplementary material. In GEP, we generally use 300 to 500 rounds of “extend” followed by only 50 rounds of “fill” because simulations showed that holes in behavior domain were always small, rare or even absent. In the illustration of Figure 1F1, the example goal is on the periphery of the behavior domain (see red cross). The closest behavior is then searched and its associated parameters are slightly modified (random function) to produce a new parameter set (New parameters). We choose a random policy in order not to introduce bias in the setting of new values for parameters. Because the policy used by GEP for parameter changes was a random function, this GEP was named rGEP. After a simulation is run (Figure 1F2), if the produced movement fulfils the acceptance criteria, it is stored in the behavior domain [see trace (3) in Figure 1F2], otherwise, it is rejected [traces (1) and (2)]. Acceptance criteria were as follows: (i) the produced movement should fit a minimum jerk angular kinematics (Friedman and Flash, 2009); (ii) there should be no oscillations in preparatory and post movement phases; (iii) the movement amplitude should be in the range (10–110°); (iv) no co-contraction should be present during the preparatory phase (controlled by SET commands, see below) and during the stabilized phase after movement (launched by GO commands) (see Methods 11 for reasons of this choice). A cost function ensured valid movements fulfill acceptance criteria (see Methods 6).

Because rGEP needs valid movements to progress, the first step, was to find initial valid movements. In order not to interfere with the design of the commands, these initial valid movements (termed seeds) were found via a state of the art optimization method covariance matrix association evolution strategy (CMAes) (Hansen et al., 2003). Briefly, a desired behavior (a movement template) was given to the optimization method that worked on the parameters of the SET and GO commands to find the result that best fitted the desired movement template (see next paragraph for more explanations). During this process of optimization, any valid movement produced was stored and could be used as a seed.

### 2.5 Optimization method used to get first behaviors (seeds)

Covariance Matrix Adaptation evolution strategy (CMAes) is an evolution algorithm developed by Hansen et al. (2003). It works with a gradient descent aiming to minimize a cost function. This evolution algorithm is based on the principle of biological evolution. This means that for each generation (iteration), new individuals are generated from variation, in a stochastic way, of the current generation. To initiate the next generation, individuals with the best scores (minimal value of the cost function) are selected to become the parents of the next generation. Following this rule, individuals of the successive generations get closer to the best results.

In the evolution algorithm, the solutions that represent the new generations are issued from multivariate-normal distribution based on a variance value (*sigma*) and the covariance matrix of the preceding best solutions. This allows the stochastic changes made to the parameters to be oriented in the direction of the solution. Moreover, the global step-size parameter *sigma* is adapted every iteration using cumulative step-size adaptation (CSA), so it can increase on broad, exploratory moves and decrease as the search converges. σ_k_ scales the overall search distribution [ $N(m_{k},\sigma_{k}^{2}C_{k})]$ , controlling how far new candidate points are sampled around the mean $m_{k}$ . A larger σ_k_ means more global exploration, while a smaller σ_k_ means local, fine-grained search near the current mean.

In the CMAes optimization procedure, some parameters have to be given before the simulation was launched including the *limits* of exploration and the *sigma* value. If the *limits* of exploration were too large, new individuals could be generated too far from each other and the optimal solution might not be reached. A small *sigma* will create a new generation not so different from the previous one and a large one will bring more changes. All optimization series were initiated with a small *sigma* (0.005) and *limits* of exploration covering 100% (i.e., between 0 and 1 for all normalized parameter values). *Sigma* and *limits* were given to CMAes at initiation of optimization process. CMAes was used to uncover single valid movement, which rGEP used as initial target to search the behavior domain (for an explanation of why, in this second phase, CMAes was not used, see Discussion section 7: Interest of rGEP compared to CMAes in the search for behavior domain).

### 2.6 Cost function used to select valid movements

For each movement produced, a cost function estimated the proximity of movements produced by the simulation to human movement. As normal human movements follow a template based on the minimum jerk model (Flash and Hogan, 1985), the cost function calculates the distance (mean square error) between the produced movement and this adapted template (see below) (Figure 1E), with the addition of the penalty for the co-contraction (Equation 1). This term was added after a first exploration of the method, which led to systematic non physiological co-contraction during the preparation period and during the maintained final position. The result was considered valid when the *Cost* value was less than 1.


$$\begin{array}{l} \text{Cost}=\sum_{t=2}^{t=10}{\left(T_{\text{desired}}(t)-T_{\text{actual}}(t)\right)}^{2}+\text{Penalty}\_\text{Prep}\\ +\text{Penalty}\_\text{Main} \end{array}$$ (1)

With *t* representing the different sampled times of the simulation (dt = 10 ms).

Building of the minimum jerk template adapted to each produced movement. For each movement, the algorithm measures its amplitude and estimates its duration by an iterative process during which minimum jerk templates are elaborated from these two parameters, and the fitting of the movement to the template is calculated. Once the best fit is obtained, the cost function was calculated according to Equation 1. Note that this process is not biologically plausible, but it is a methodological tool to obtain minimum jerk movements which are biologically relevant.

#### 2.6.1 Eliminating co-contraction

In order to avoid solutions in which co-contraction occurred during the preparatory phase (or after movement stabilization), co-contraction penalty was added in each of these phases. *Penalty_Prep* was calculated during the preparatory phase (start = 2 s, end = 5 s), and *Penalty_Main* during the maintained position phase (start = 7 s, end = 10s). The movement phase was not included because complex interactions may occur due to the dynamics of sensorimotor interactions. The penalty for co-contraction was calculated as indicated in Equation 2.


$$\begin{array}{l} \text{Penalty}\_i=\frac{1}{\mathrm{end}-\text{start}}\\ {}*(\int_{t=\text{start}}^{t=\mathrm{end}}(\mathrm{VM}N_{\text{flex}}-\text{Vthr})*(\mathrm{VM}N_{\mathrm{ext}}-\text{Vthr})*\mathrm{dt})*\text{coeff}\_i \end{array}$$ (2)

in which *VMN_flex_* and *VMN_ext_* are the membrane potential of the flexor and extensor MNs, respectively, and *V_thr_* = -60 mV, is a threshold voltage under which penalty = 0.

The coefficient (*coeff_i*) was set to 100 during the preparatory phase and the maintained position after movement to make sure coactivation would be rejected in these phases.

### 2.7 Time course of the rGEP algorithm

**Figure 2:**
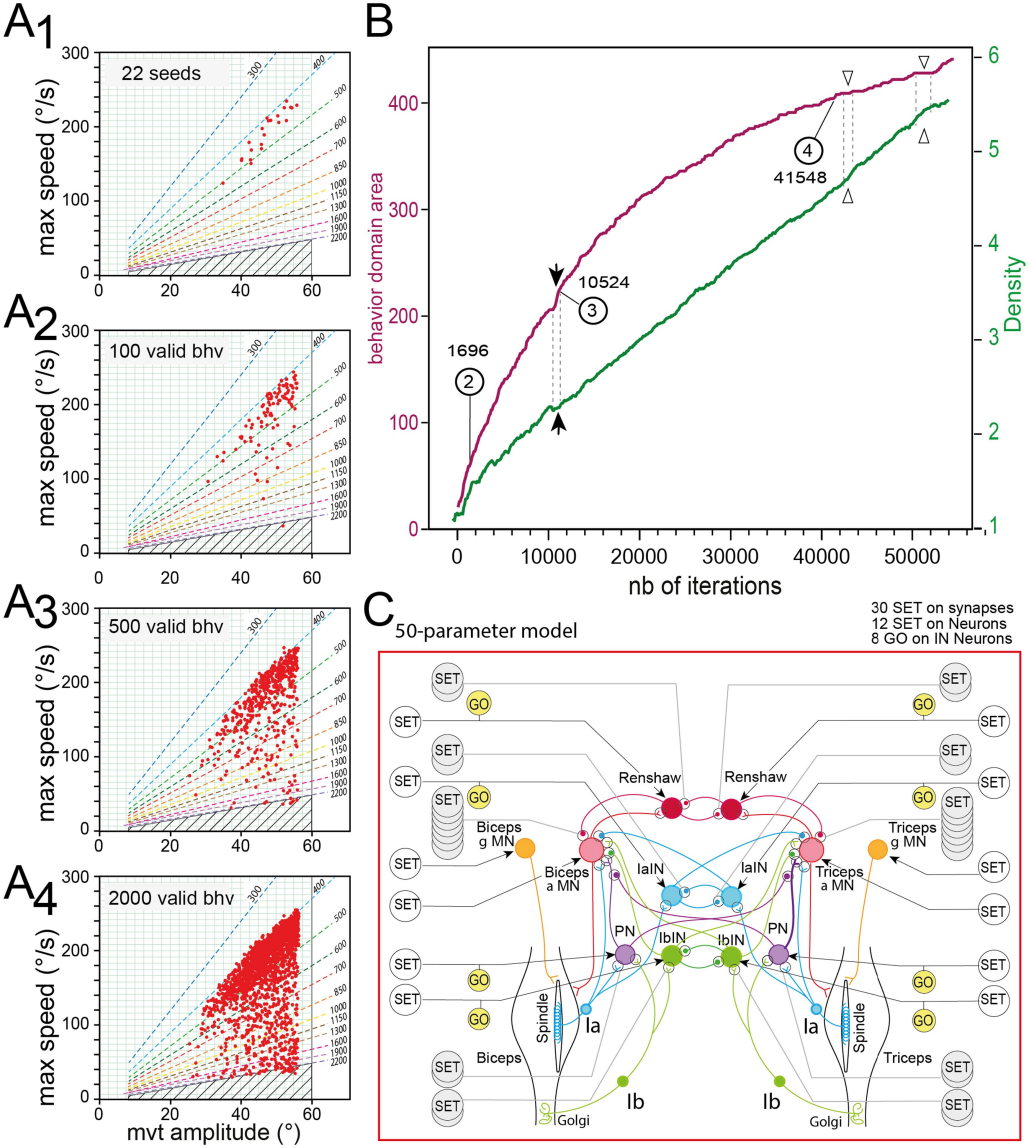
Temporal evolution of rGEP. **(A)** Progression of the behavior domain uncovered by rGEP after starting with 22 seeds **(A1)**, and extending to 100 **(A2)**, 500 **(A3)**, and 2000 **(A4)** valid movements. Same representation as in Figure 1D. **(B)** Time course of behavior domain area expansion [i.e., number of squares containing at least one valid movement in the green grids presented in **(A1–A4)** presented in violet trace]. The mean density of valid movements in the behavior domain area is presented in green trace. The numbers 2, 3, and 4 correspond to the behavior domains representations shown in **(A2–A4)**. **(C)** Diagram of the 50-parameter model.

An example of rGEP time course is presented in Figure 2, on the same model presented in Figure 1 (see also inset network diagram in Figure 2). Before rGEP starts, valid movements (seeds) must be given to the algorithm (see below). In the example presented in Figure 2, 22 seeds were given (Figure 2A1).

Four pictures of the behavior domain extension are presented starting from 22 seeds (Figure 2A1), and extending to 100, 500 and 2000 valid movements (Figures 2A2–A4), corresponding to 1,696, 10,524 and 41,548 runs of simulation, respectively. As the rGEP progresses, the behavior domain extends (Figure 2B, violet trace, *behavior domain area*). The behavior domain area was quantified as the number of cases of a grid, defined in the behavior domain (see Methods), which contain at least one valid movement (see green grids in Figures 2A1–A4).

During the progress of rGEP, the borders of the behavior domain progressively extend. In the last rounds of extend, the borders do not progress much, but the density of valid movements located at the periphery of the behavior domain progressively increase. Finally, if there was enough extend rounds, the density of behaviors on the borders are dense enough to define a clear border line to the behavior domain. While the borders of the behavior domain get more and more precise, there were still some empty central zones when the process was stopped after 300 “extend” rounds and 50 “fill” rounds (totalizing 54,326 runs). Note that there is no direct relationship between the number of extend rounds and the number of runs, because the number of runs in a given round depends on the size of the behavioral domain at the time the round is launched. Note also that when the rGEP was stopped, the process was not complete as indicated by the persistent increase of behavioral domain area (violet trace) during the last 2000 runs (Figure 2B). Some empty zones still persist after the 300 extend rounds and 50 fill rounds. This domain was then completed by adding four new rGEP with 300 expands and 50 fills (starting from new seeds each time) totalizing 178,601 runs [see below “Behavior domain of the complete (50-parameters) model”].

We can also see that the progression of rGEP is not linear. Indeed, several transient plateaus were observed (see open arrow heads in Figure 2B) on the behavior domain area curve (violet trace). During such blockage of exploration, valid movements were found in the already discovered behavior domain, resulting in a transient increase of the mean density (see methods) of valid movements found in the behavior domain (green curve in Figure 2B). The situation unblocks when a new valid movement is found that occupies an empty case in the grid, and allows new valid movements to be found from its parameter set, resulting sometimes in an abrupt increase of the behavior domain area (see filled arrow heads in Figure 2B) while the progression of mean density slows down (see green trace, in Figure 2B). As the behavior domain extends (Figures 2A,B), the parameter domain also evolves.

### 2.8 Modelling coding properties of muscle spindle

**Figure 3:**
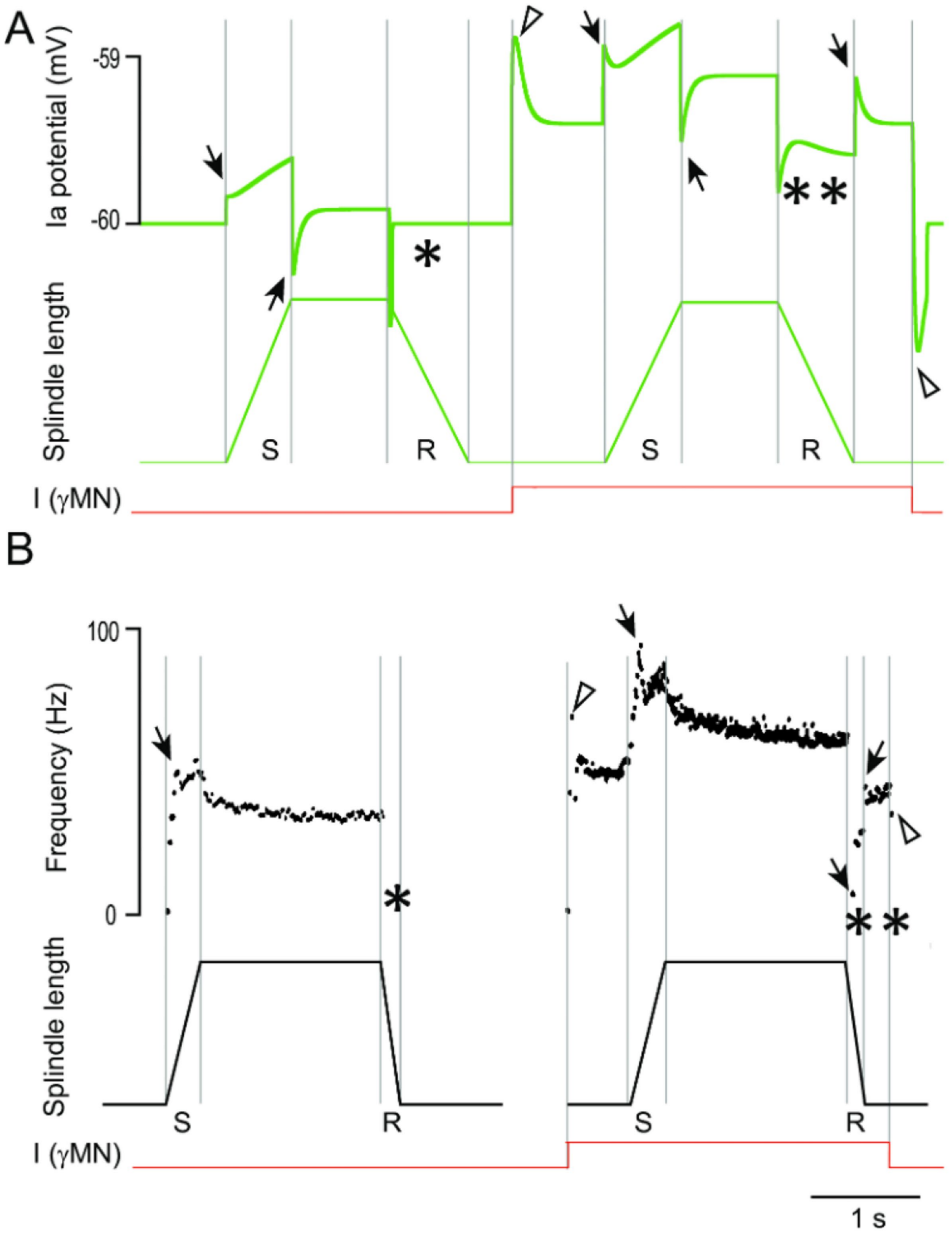
Muscle spindle coding properties. **(A)** Modeled extensor muscle spindle activity during stretch (S) and release (R) ramps, without (left), and with (right) activation of the gamma motoneuron (I_γ_MN). **(B)** Electrophysiological recording of the Ia fiber during the same imposed movements as in **(A)** adapted from Boyd et al. (1977) with permission from John Wiley and Sons. Note that, in both simulation and physiological recording, the first release of the spindle does not give any response (see *) due to lack of tension of the spindle. However, the activation of the γMN allows the spindle to give an answer during the second release (see **). Note also that in **(A)**, the “acceleration” component produced by the serial spring velocity (in the Hill model) is responsible for the transient activation at each movement change (arrows), while the “tonic” component produced by the tension of the spindle (in the Hill model) produces an exponential decay shape. Transients are also produced at onset and offset of the γMN stimulation. These features are present in **(B)** except for transients at the end of stretch movements.

Muscle spindles were added to each muscle. In AnimatLab (see Methods), muscle spindles have the same “attachments” as the muscles. Their tension-length ratio, maximum force capacity, and viscoelasticity are based on the Hill muscle model (Scovil and Ronsky, 2006). To assess the quality of this neuro-musculoskeletal model, imposed movements of flexion and extension were simulated, while muscle spindle activity was recorded (Figure 3A). Our first work was to adjust by hand the muscle spindle parameters that controlled their coding of movements so that their response were comparable to electrophysiological data from the literature (Boyd et al., 1977) (Figure 3B), in the absence and in the presence of activation of a Gamma MN.

Note that the AnimatLab platform proposes a single type of gamma command that is a mixture of gamma static and gamma dynamic properties. Therefore, in all simulations we used this single type of gamma MN (see Discussion F for more details).

### 2.9 Computation of EMG signal

In the model, muscles received continuous input from non-spiking motoneurons, as long as the membrane potential of motoneurons was over the threshold for synaptic transmission. This threshold was set to -60 mV to ensure that no contraction would occur as long as the muscle does not receive any input from MNs (i.e., as long as MN potential stays below this threshold value). The muscle membrane potential was continuously monitored. At rest it was set to -60 mV, a potential at which no force is produced in the muscle (see Stimulus-Tension parameters in Table 2). During supra-threshold activity of MNs the continuous synaptic transmission between MNs and muscle resulted in a depolarization of muscle membrane, and force was produced. In this model, due to the absence of spikes, the EMG signal was directly assessed as the muscle membrane potential.

### 2.10 Identification of triphasic pattern

Identification of triphasic pattern relies on a precise identification of Flexor peaks (AG1 and AG2) and extensor peak (ANT). Thanks to our choice to use non-spiking neurons, the membrane potential of muscles evolves without spikes and is used as an EMG envelope of classical spiking EMG signals. Moreover, because there is no MN activity prior to movement, and no activity during the maintained position after the movement phase, muscle potential is at −60 mV before and after movement. The three peaks (AG1, AG2 and ANT) are detected and measured using an algorithm (see repository of python scripts made available at the address given in the Data availability statement) that search for all maxima present in biceps and triceps membrane potential recordings. Between Maxima, minima are then searched. These operations are facilitated by the fact that signals start from −60 mV (before movement) and go back to −60 mV (after movement). Therefore, any muscle activity requires the presence of a peak of muscle membrane potential over −60 mV. The algorithm detects even the smallest peaks. Triphasic patterns are then analyzed from these peaks data obtained on each movement. The specific triphasic search algorithm collects all movements in which two biceps peaks are present and a triceps peak is present between the two biceps peaks. Note that there can exist a silent period between the two bicep’s peaks or not, and that consequently various degrees of overlapping were observed between biceps’ and triceps’ peaks.

### 2.11 Why use absence of co-contraction in preparatory and final position maintenance?

In a preliminary approach in which co-contraction was allowed the GEP found many movements with co-contraction. Notably, many fast minimum-jerk movements were achieved with a strong co-contraction during the preparatory phase (due to the SET commands), and a sudden stop of triceps activity resulting from the GO command. Although this kind of strategy has been observed in animals [such as in locust jump, see Cofer et al., 2010], it has not been reported in human elbow flexion. As this strategy represented a majority of solutions found by rGEP that would have masked other, more physiological solutions, we have decided not to allow co-contraction during the preparatory phase. Concerning endpoint stability at the end of the movement, since our movements are performed in the absence of gravity [a situation comparable to the experiments of Hallett et al. (1975)], there was no need for maintaining muscles activities in this situation, although a slight residual tonic activity is often observed. For simplicity, we have decided not to consider such residual tonic activity, and therefore not to allow co-contraction at the maintained position phase. Moreover, our aim was not to describe all movements (with a minimum jerk profile) that could be produced by the model, but to test if sensorimotor spinal networks could elicit triphasic commands in the absence of any dynamics in the descending commands. The absence of co-contraction (except during movement execution) has the advantage to simplify the approach, making easier the detection of peaks of activity in biceps and triceps (see above chapter 10).

## 3 Results

### 3.1 Goal exploration process explores the capacity of spinal sensorimotor circuits and their step commands to produce behaviors

In this section, we provide a brief overview of the neuromechanical models and the process (rGEP) used to explore their behavioral capabilities.

#### 3.1.1 Behavior domain of the complete (50-parameters) model with both Ia and Ib afferents and associated network

**Figure 4:**
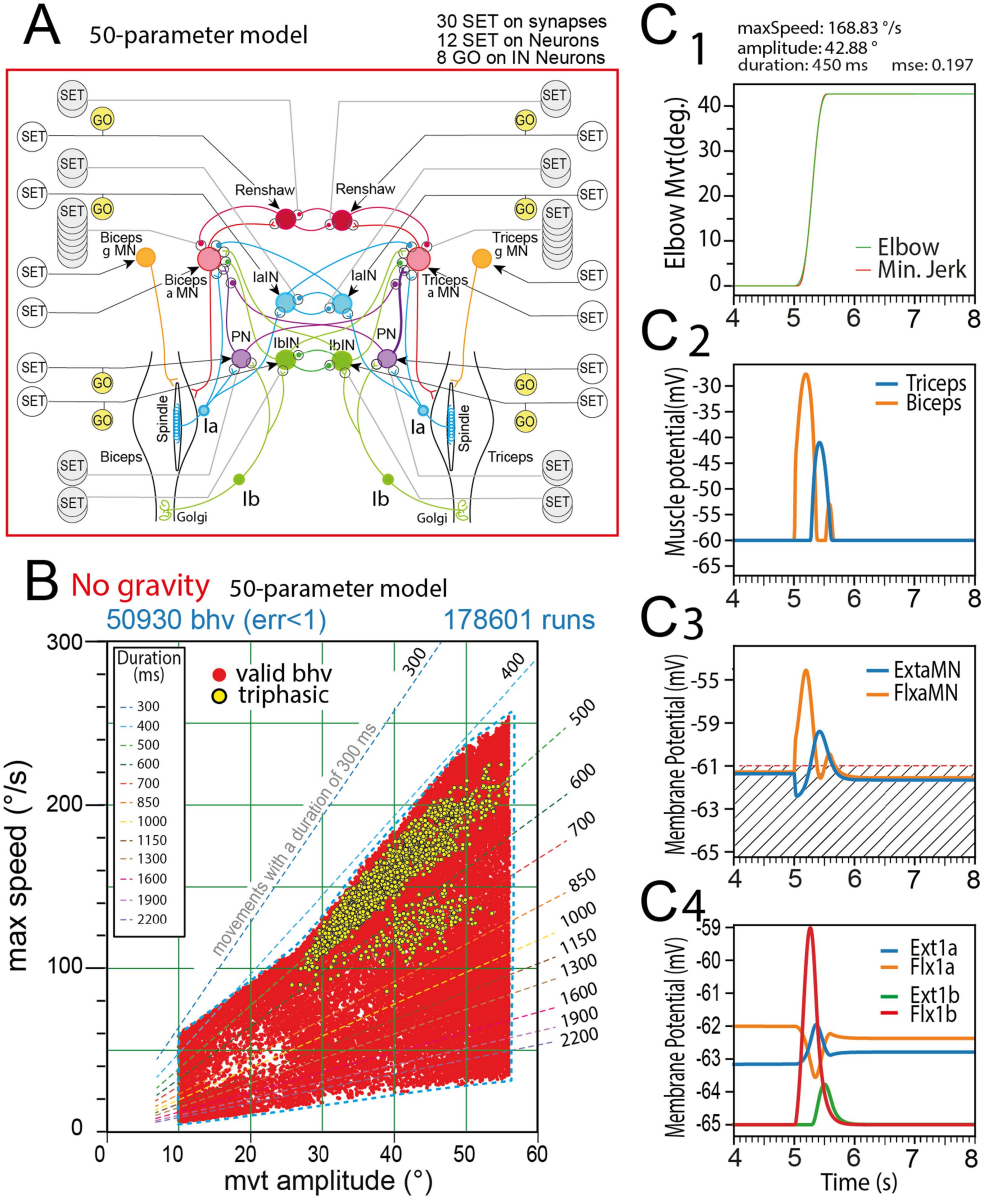
Movements produced by rGEP with the 50-parameters model. **(A)** Sensorimotor spinal network of the 50-parameters model. **(B)** Behavior domain uncovered by rGEP on this model. The perimeter of the domain is outlined with a blue dashed line. Behaviors with triphasic pattern are presented as yellow circles. **(C)** Representative example of one valid movement in the behavior domain **(C1)**, with time course of membrane potential of biceps and triceps muscles **(C2)**, Flexor and Extensor MN membrane potential (see FlxαMN and ExtαMN in **C3**), and 1a and 1b afferent neurons potential (see Flx1a, Ext1a, Flx1b, Ext1b in **C4**). Striped area in **C3** indicates MN under-threshold potentials, under which no muscle response was observed.

The behavior domain found for the 50 parameters model by the rGEP is presented in Figure 4B. It was obtained by cumulating 5 rGEP explorations, each composed of 300 “extend” rounds and 100 “fill” rounds, and totalizing 178,601 runs, in which 50,930 movements were valid. The behavior domain is defined by two parameters of the produced movements: maximal speed (ordinate) and movement amplitude (abscissa). A third characteristic of movements (movement duration) is presented on the graphs by oblique lines labelled with the represented duration (in ms). Only physiologically plausible movements have been retained (slow movements the duration of which was > 2 s have been rejected – see striped area). Only movements with an amplitude in the range [10°, 110°] have been retained, to avoid too small movements and too large movements that could reach the flexion limits. However, with both Ia and Ib sensorimotor circuits complete, this model could not perform movements larger than #55° at a max speed of #250°/s and movement durations not less than 400 ms. Note for each amplitude, a great variety of speeds was found.

A representative example of a movement (amplitude 42.88°) is given in Figure 4C1. Note the profile of the movement that fitted a minimum jerk profile with small MSE value (0.197). Several other variables are also presented for this example: Membrane potentials of muscles, MNs, and sensory neurons. At the initiation of the movement, the membrane potential recorded in the biceps reached > − 30 mV within 200 ms, and then decayed to its resting potential in 200 ms (Figure 4C2). The membrane potential recorded in the triceps increased at the end of the rapid decrease of biceps potential peak, and reached −40 mV at *t* = 540 ms, and then decreased in the next 200 ms, at the end of the flexion movement. Note the small second depolarizing peak in the biceps while movement stops.

The activity of alpha MNs present transient activities during the movement phase: the biceps MN (*FlxαMN*) presents two peaks of depolarization, the second being smaller, and an absence of activity between the two peaks, during which the triceps MN (*ExtαMN*) presents a peak of activity (Figure 4C3). Once the flexed position is reached, the activity of both alpha MNs totally vanishes. During the movement phase, the proprioceptive feedback from Ib afferents was much larger than that of the Ia afferents (Figure 4C4). The shape of Ib activations was also different from that of Ia afferents: during the rapid flexion movement, the Ib activity of the flexor muscle (*Flx1b*) present a large peak during the acceleration of the flexion movement, in accordance with its role in sustaining the flexion movement via the Flexor PN excitatory circuit (see Figure 4A); this initial *Flx1b* peak is followed by a smaller peak of the extensor 1a activity (*Ext1a*) that contributes to slow down the flexion movement due to its excitatory effect on the extensor αMN (see Figure 4A); this slowing effect is prolonged by the delayed increase of *Ext1b* activity that contributes to the activation of the extensor αMN via the PN interneuron of the extensor circuit (see Figure 4A). Note that during the flexion movement, the activity of the Flx1a presents a trough. Note also the small peak of Flx1a just before stabilization at −62.38 mV.

The time course of 1a and 1b afferent activities (larger Flx1b peak followed by a smaller Ext1a peak, and by a delayed peak of the Ext1b activity, concomitant with the recovery of Flx1a trough) were regularly observed in the 50-parameter model, with some small variations all along its behavior domain. This first study will serve to compare the capacities of other models issued from this one, but lacking some neural elements. To facilitate comparison, the perimeter of the behavior domain of the 50-parameter model (see dashed blue line in Figure 4B) will be superimposed on their behavior domains.

#### 3.1.2 Behavioral domain produced in the absence of Ib feedback (with central cross connections)

**Figure 5:**
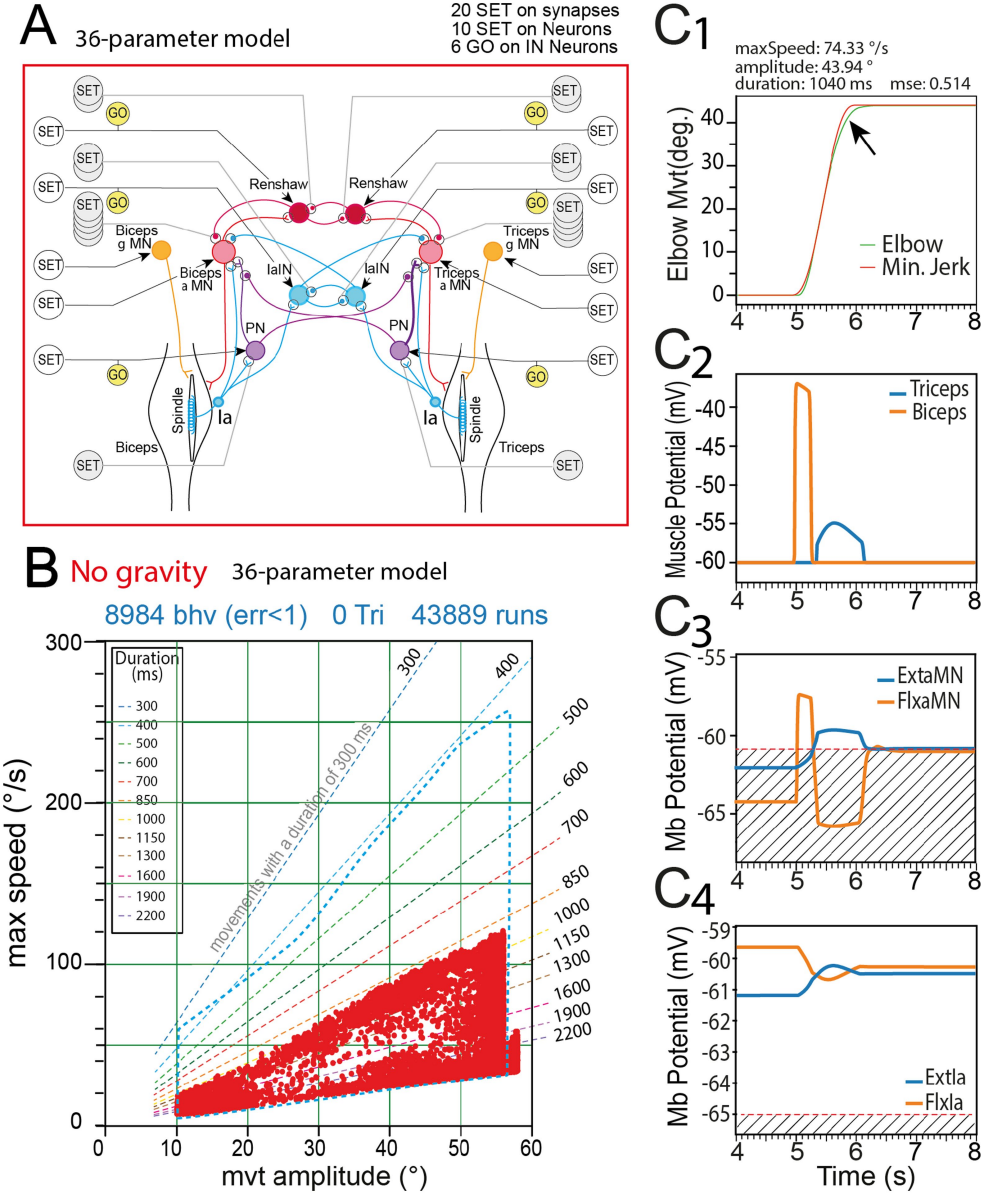
Movements produced by rGEP with the 36 parameters model. **(A)** Sensorimotor spinal network of the 36 parameters model. **(B)** Behavior domain uncovered by rGEP on this model. For comparison, the perimeter of the domain of the 50-parameter domain is represented with blue dashed line. **(C)** Representative example of one valid movement in the behavior domain **(C1)**, with time course of membrane potential of biceps and triceps muscles **(C2)**, Flexor and Extensor MN membrane potential (see FlxαMN and ExtαMN in C3), and 1a afferent neurons potential (see Flx1a, Ext1a in **C4**). Striped area in **C3** indicates the potentials under which no muscle response was observed. Striped area in **C4** indicates under threshold activity.

In order to study what proprioceptive feedback was necessary to produce valid movements, in a first step, we suppressed the Ib input, the Ib INs and their synapses (Figure 5A). When rGEP was run on this model, the resulting behavior domain was much smaller than the one obtained with the complete spinal network (compare Figures 4B, 5B), due to the maximal speed being greatly reduced, and hence, all movement durations were longer than 850 ms.

Activities of muscles, MN and muscle spindles feedback (Figure 5C) were different from that of the complete model (Figure 4C) for comparable movements. A representative example of a movement (amplitude 43.94°) is given in Figure 5C1, with a maximal speed of 74.33°/s. However, the fitting to a minimum jerk template (MSE = 0.514) was not as good as in the complete model. This was likely due to the slowdown at the end of movement (see arrow in Figure 5C1) being much more progressive than in the complete model. The biceps muscle peak of depolarization (−37 mV) (Figure 5C2) was slightly smaller than that observed for the complete model (-28 mV). Strikingly, in this model deprived of Ib afferent circuits, the second peak of Flx muscle activity and Flx Alpha MN activity was never observed (Figures 5C2,C3). This second peak was also absent from the Flx1a activities (Figure 5C4).

#### 3.1.3 Behavioral domain produced in the absence of Ib feedback (without central cross connections)

**Figure 6:**
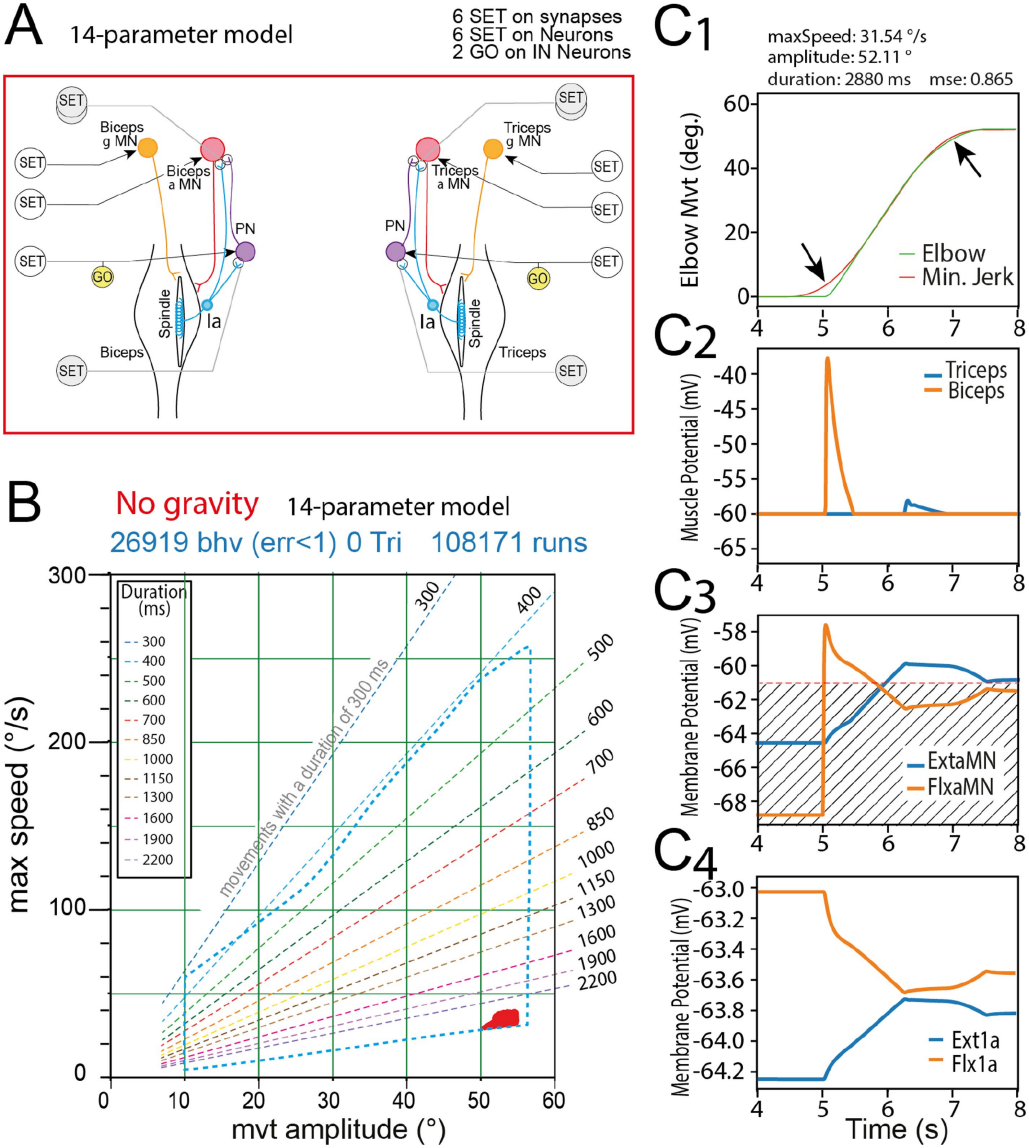
Movements produced by rGEP with the 14-parameters model. **(A)** Sensorimotor spinal network of the 14 parameters model. **(B)** Behavior domain uncovered by rGEP on this model. For comparison, the perimeter of the domain of the 50-parameter domain is represented with blue dashed line. **(C)** Representative example of one valid movement in the behavior domain. Striped area in **C3** indicates the potentials under which no muscle response was observed.

To go further in the analysis of the involvement of Ia proprioceptive feedback in the production of minimum jerk movements, we drastically reduced the sensorimotor network, by suppressing all relationships between extensor and flexor parts of the network, as well as all interneurons except propriospinal INs. The resulting spinal network is the 14-parameters simplified network presented in Figure 6A with the same design as other models presented in Result section. Interestingly, this extremely simplified spinal network, reduced to Ia proprioceptive inputs connecting their homonymous MN either directly or via their propriospinal IN (PN), was still capable of producing some valid movements (Figure 6B). The resulting behavior domain was, however, extremely smaller than the one of the 36-parameters mode (no movement shorter than 2,200 ms were produced and maximal speed was under #40°/s). A representative valid movement example (amplitude 84.29°) is given in Figure 6C1. Note that in most of the valid movements produced by this model, the fitting to a minimum jerk template was not as good as in the complete model (here MSE = 0.865). Moreover, the second peak in Flx force, Flx alpha MN and Flx1a activities was never observed (Figures 6C2,C3).

#### 3.1.4 Behavioral domain produced in the absence of Ia feedback with central cross-connections

**Figure 7:**
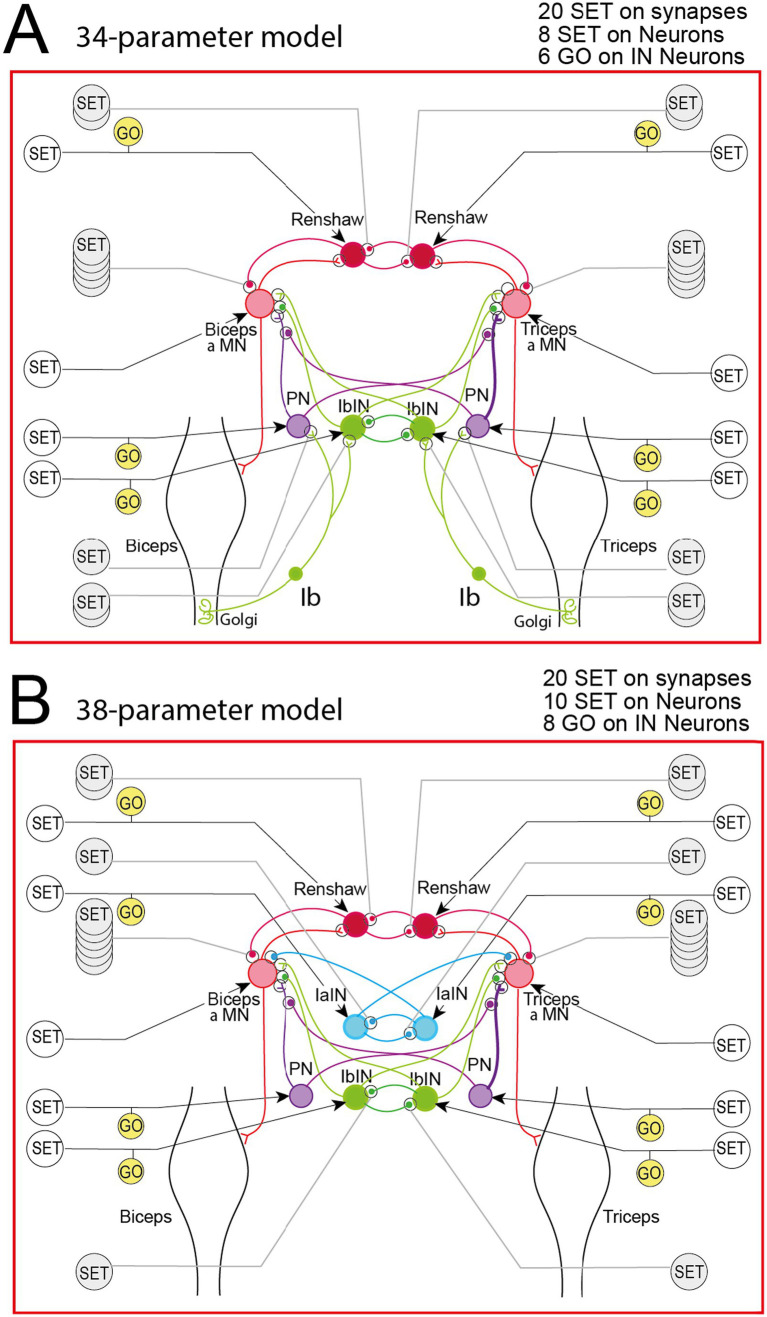
34- and 38-parameters models. **(A)** Sensorimotor spinal network of the 34 parameters model. **(B)** Sensorimotor spinal network of the 38 parameters model. The very slow movements produced by these models did not meet criteria for valid movements.

We then applied the same method to test Ib afferents involvement in the production of valid movements via rGEP. Starting from the complete (50-parameters model), we suppressed Ia afferents, Ia INs and their synapses (Figure 7A). This Ib network could not produce any valid movements.

#### 3.1.5 Behavioral domain produced in the absence of any feedback but with all INs

Because reciprocal connections seemed important to perform valid movements, we tested if complete spinal network with all reciprocal connections but deprived of any proprioceptive feedback (Figure 7B) could produce valid movements. However, no valid movements were found with this model.

### 3.2 Analysis of triphasic patterns in the various models

By design, valid movements found by rGEP followed the minimum jerk rule. However, as we will see, this rule does not imply conformity with a triphasic command. Triphasic command is defined from the EMG recordings of two antagonistic muscles that were involved in a rapid movement around a joint (Wachholder, 1928; Angel, 1974; Wadman et al., 1979; Brown and Cooke, 1981). Typically, the agonist muscle produced two bursts of activity: one (AG1) at the onset of movement and one (AG2) at end of the movement. The amplitude of the initial agonist burst (AG1) was larger than that of the second (AG2) (Bullock, 1992). In between the two agonist bursts, an antagonist burst (ANT) is present (Sanes and Jennings, 1984), the role of which is to slow down the movement speed so that, ideally, the speed is 0 when target angle is reached. However, in rapid movements, if the activity of the antagonistic muscle is too large, the movement goes back. The asymmetrical rise- and fall-times inherent to muscle activation may also induce a prolonged effect of ANT. These effects are counteracted by AG2. This excess of ANT activity (in amplitude and/or in duration) and the second burst of the agonist (AG2) produce a small oscillation of the joint angle. Such small oscillations at the movement end were accepted as long as the error (MSE) calculated between the movement and its minimum-jerk profile was less than 1. Movements with too large end oscillation (see Figure 1F, right, profile (1)) were rejected.

**Figure 8:**
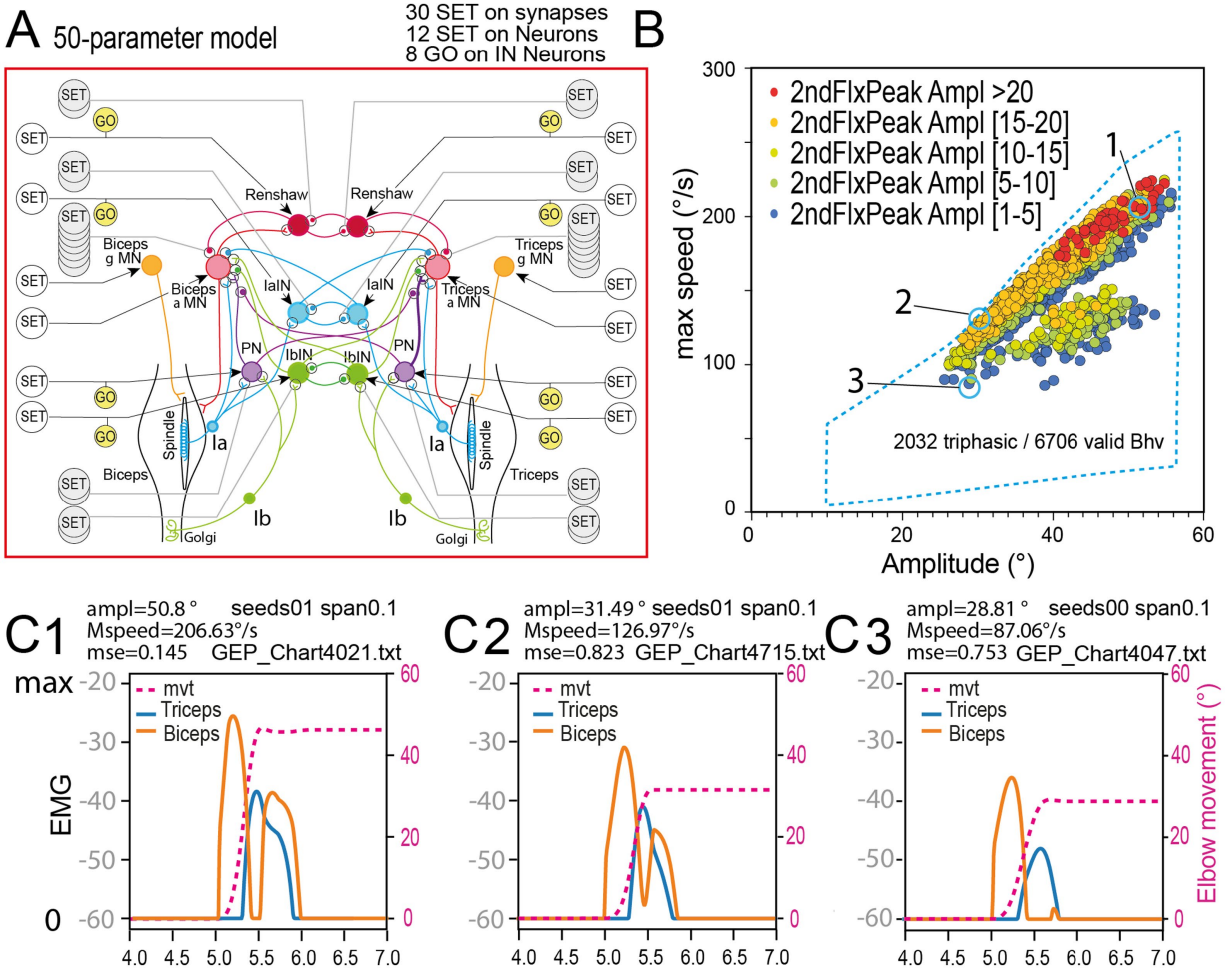
Presence of triphasic pattern in the behavior domain of the complete 50 parameter model. **(A)** Sensorimotor spinal network of the 50-parameters model. **(B)** Presence triphasic pattern in the behavior domain uncovered by rGEP on this model. As can be seen triphasic pattern seems noticeably regrouped in the upper right part of the behavior domain, which contains the faster/larger movements (see blue dashed lines for the limits of the 50-parameter behavior domain). **(C)** Examples of triphasic pattern for large (50°, **C1**), medium (42°, **C2**), and small (35°, **C3**) movements. These 3 examples are identified in B with numbers 1, 2, and 3.

In order to apply the definition of the triphasic command, we have used the electrical activity of muscles. However, since all models used in this study were based on non-spiking neurons (and hence non-spiking muscle), we used the non-spiking muscle activity to analyze triphasic command occurrences. In order to illustrate EMG profiles, for each model, we give examples of EMGs produced during large fast movement (#50°; #200°/s), and during medium amplitude movements (#30°) either rapid (#130°/s), or slow (#90°/s) (see Figures 8C1–C3).

#### 3.2.1 Triphasic commands with the complete (50-parameters) model

The occurrence of triphasic commands with AG1, ANT, AG2 peaks of activity were only observed with the 50-parameters model (see the full network in Figure 8A). Triphasic patterns with two Flexor peaks, separated by a silent period during which an Ext peak was present, seems noticeably regrouped in the upper right part of the behavior domain, which belongs to faster/larger movements produced by the 50 parameters model (Figure 8B). Three examples of triphasic pattern are given in Figures 8C1–C3, for large fast movement (#50°; #200°/s; Figure 8C1), medium fast movement (#30°; #130°/s; Figure 8C2) and medium slow movement (#30°; #90°/s; Figure 8C3). Note that these movements were valid because they followed a minimum jerk profile (their MSE were 0.145, 0.823 and 0.753, respectively).

Here we represented only the movements associated with a triphasic pattern. However, such movements associated with a triphasic pattern represent only a fraction of the valid movements found by rGEP. For example, during a rGEP session on the 50-parameters model, 18,983 runs were made resulting in only 6,706 valid movements. Among these, 2032 movements (30.3%) presented a triphasic pattern. Indeed, more than 2/3 of movements found by rGEP consisted in a single Flx EMG peak followed by a single Ext EMG peak. In Figure 8B, triphasic movements occupy only a fraction of the behavior domain (fast and large movements) in which they represent a large majority; but as movements become slower, the amplitude of the second peak gets smaller (see color scale in Figure 8B), and bellow 80°/s no triphasic movement occurs.

#### 3.2.2 Triphasic commands with the other models

In the other models, we could not find any valid movement with a triphasic pattern. Even in the 36-parameters model (complete model deprived of the 1b afferent and Ib Interneurons), there were valid movements very close to minimum jerk (MSE < 0.05) but the second Flx EMG peak was always absent. Nevertheless, we could find very rare pseudo triphasic patterns. They were rejected either because the second peak was so small that it did not elicit any force in the biceps, or because their profile did not meet the minimum jerk criterium (MSE > 1). Moreover, all of these pseudo triphasic profiles were found in slow movements (data not shown).

#### 3.2.3 Elaboration of triphasic commands in spinal sensorimotor networks

**Figure 9:**
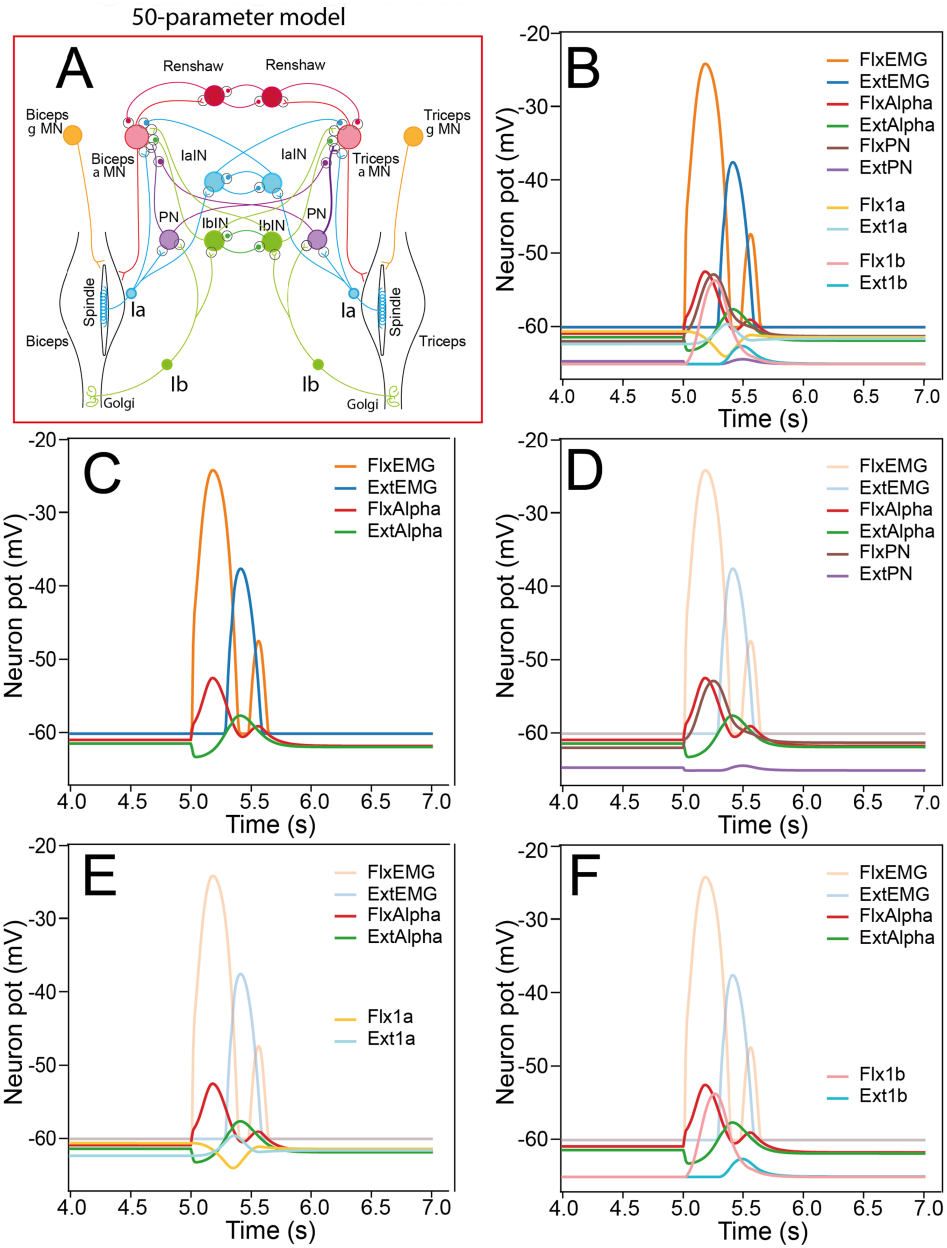
Activity of spinal neurons during triphasic pattern with the complete 50 parameter model. **(A)** Sensorimotor spinal network of the 50-parameters model. **(B)** Activity of Alpha MNs, PN interneurons, 1a and 1b sensory neurons during a triphasic command. **(C)** Relationship between biceps EMG (FlxEMG), triceps EMG (ExtEMG), biceps alpha MN (FlxAlpha) and triceps alpha MN (ExtAlpha). **(D)** Relationship between propriospinal interneurons (FlxPN and Ext PN) and their corresponding MNs (FlxAlpha and ExtAlpha). **(E)** Relationship between 1a sensory afferents (Flx1a and Ext1a) and their corresponding MNs (FlxAlpha and ExtAlpha). **(F)** Relationship between 1b sensory afferents (Flx1b and Ext1b) and their corresponding MNs (FlxAlpha and ExtAlpha).

An example of triphasic commands with AG1, ANT, AG2 peaks of activity was used to examine the temporal relationships between EMG, Alpha MN activity, PN activity and sensory 1a and 1b activity (Figure 9). Note that in this example, the two flexor EMG peaks are separated by a total return to resting potential. In some other valid movements, however, a partially overlap could be observed, as was reported in physiological reports for rapid movements [see Figures 1C,D in Brown and Cooke (1981)]. Alpha MN, PN and sensory 1a and 1b represent all the spinal sensorimotor neurons that participate to the dynamical activity leading to the triphasic command (Figures 9A,B). Only gamma MNs are not represented because they only display step activation. Constitutively, FlxEMG and ExtEMG are directly dependent on the activity of FlxAlpha and ExtAlpha, respectively (Figure 9C). In the same way, the activity of FlxAlpha and ExtAlpha should depend on FlxPN and ExtPN, respectively (see Figure 9A), but the time course of the peaks observed in Alpha MNs and their corresponding PN are different (Figure 9D). The difference is small for FlxPN, the peak of which is delayed (87 ms) compared to FlxAlpha peak, but displays a similar amplitude. The peak of ExtPN is also delayed (106 ms) compared that of ExtAlpha, but its amplitude is much smaller. These mismatches can be explained because Alpha MNs receive also other input from their corresponding 1a afferents (Figure 9A). The role of Flx1a will be more precisely analyzed in the next paragraph. Note simply that Flx1a activity (yellow curve in Figure 9E) decreases during most of the FlxAlpha 1st peak. By contrast the role of Ext1a is more directly understandable because Ext1a depolarizes at the same time as ExtAlpha (compare light-blue and green traces in Figure 9E). However, ExtAlpha peak occurs later than Ext1a peak. Interestingly, this could be due to the contribution of 1b afferent via ExtPN (see Figure 9F). The large early peak of Flx1b contributes to the activation of FlxAlpha via its connection to FlxPN (compare pink and red traces in Figure 9F).

**Figure 10:**
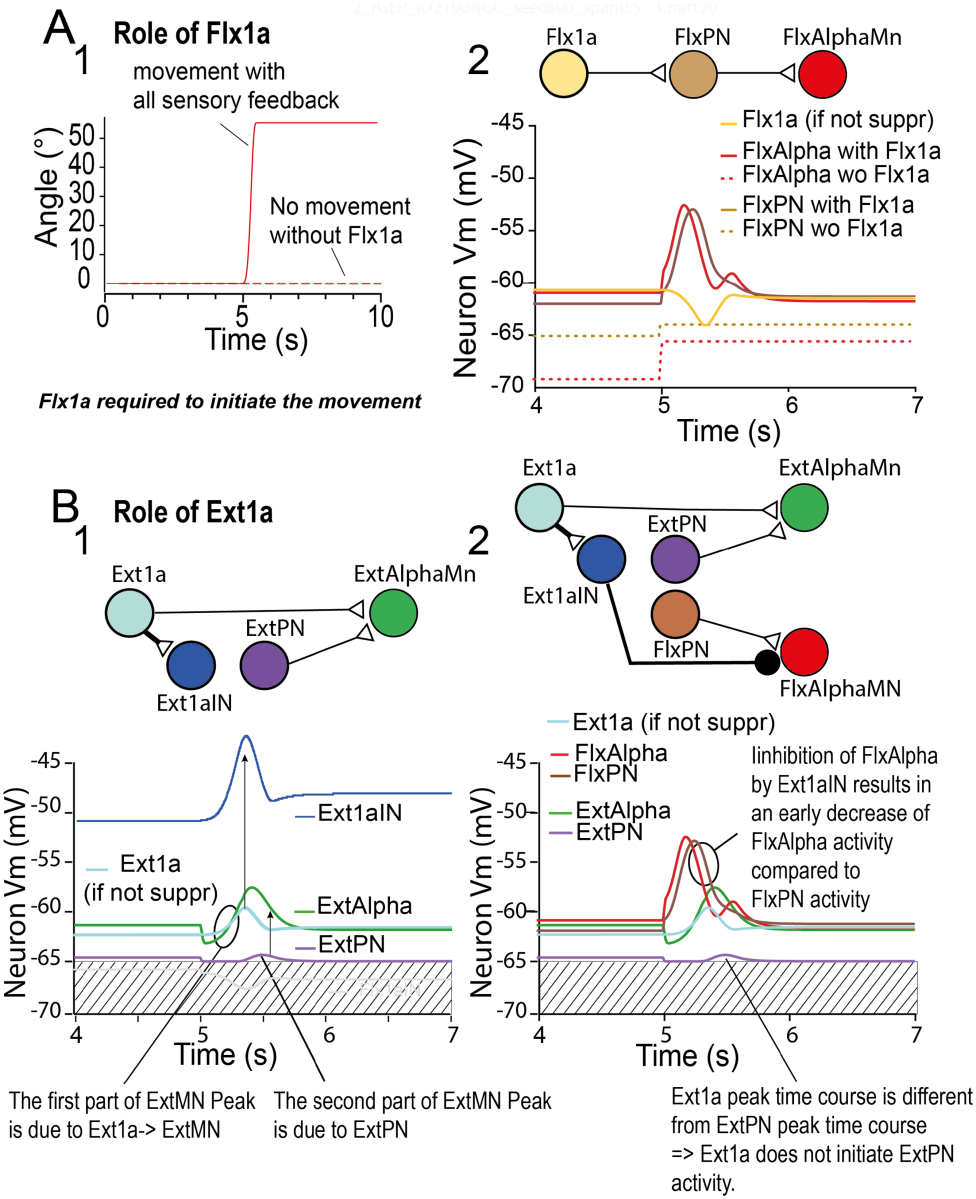
Roles of Flx1a and Ext1a. **(A)** Role of Flx1a. When the same parameter set as presented in Figure 9 was replayed with inactivation of the Flx1a afferent: no movement was observed (**A1**). The control FlxAlpha and FlxPN activities are presented in red and brown traces, respectively (**A2**). The control Flx1a activity is presented in a yellow trace (**A2**). **(B)** Role of Ext1a. When Ext1a afferent is inactivated, a full flexion is immediately produced (not shown). Therefore, we present here only the control situation. **B1**, Ext1a (light-blue trace), the activity of which starts at the onset of the preparatory phase, activates Ext1aIN (dark-blue trace – see their concomitant peaks indicated with an arrow) and ExtAlpha (green trace – see the simultaneous initiation of their peaks indicated with a circle). Note also that ExtPN (violet trace) contributes to the delayed peak of ExtAlpha (see above functional connection diagram). **B2**, FlxAlpha and FlxPN activities are presented in red and brown traces, respectively. Note that due to the activation Ext1aIN (by Flx1a), FlxAlpha terminates its 1st peak before FlxPN (see circle). A functional wiring diagram is presented above.

**Figure 11:**
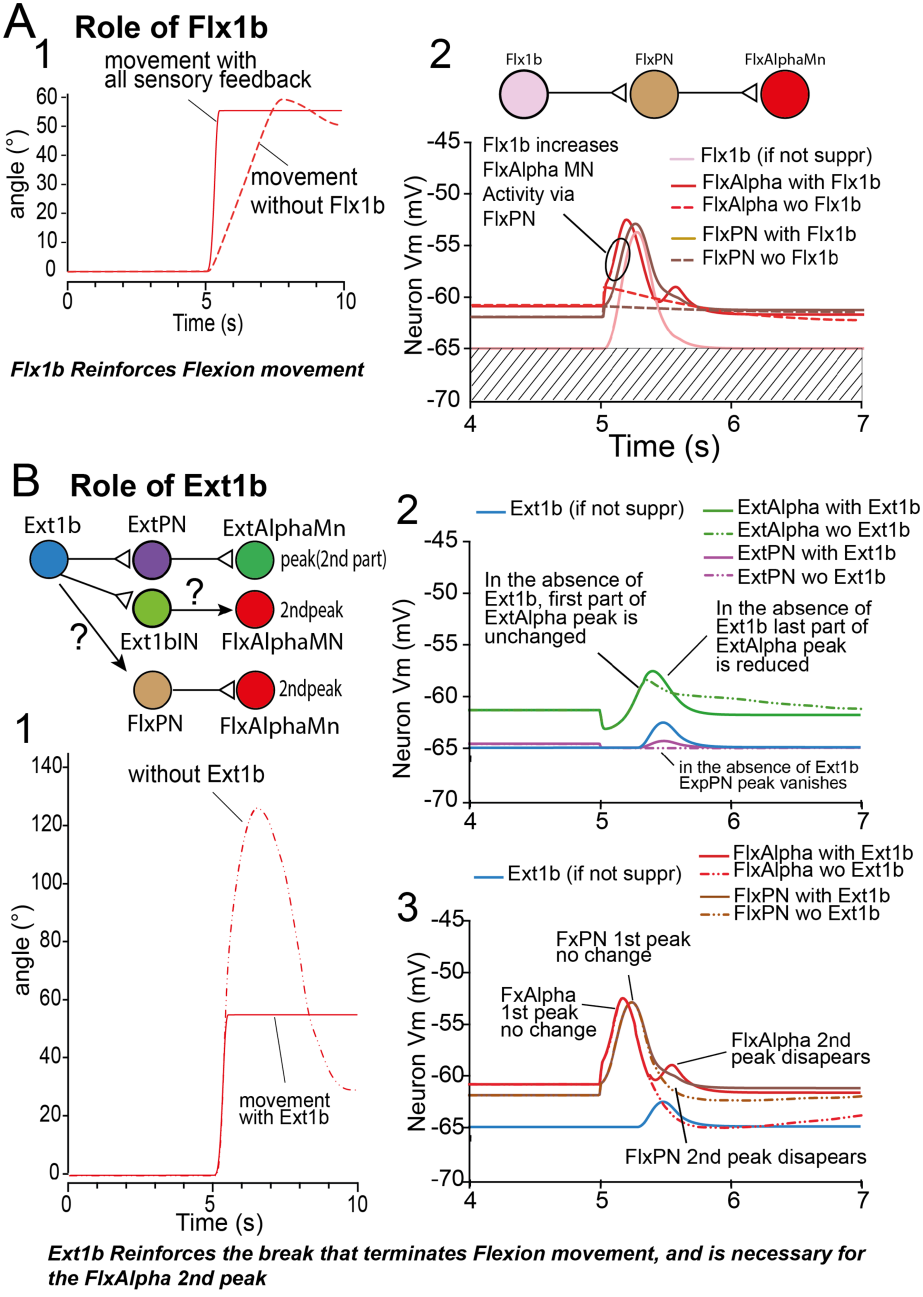
Role of Flx1b and Ext1b. **(A)** Role of Flx1b. After Flx1b afferent is inactivated, a slow movement is still produced (see dotted line), but it is unable to stabilize **(A1)**. In the absence of Flx1b activity, the FlxPN and Flx1Alpha peaks vanish (see brown and red dashed lines, respectively). **(B)** After Ext1b afferent was inactivated, a movement is still produced (see dotted line in **B1**), but with a huge overshoot. The control Ext1b activity is presented in a blue trace **(B2)**. When it is absent, ExtPN peak totally vanishes (dashed violet line in **B2**), and ExtAlpha peak is truncated (dashed green trace in **B2**). Moreover, the second FlxAlpha peak (red curve) and the second FlxPN peak (brown curve) disappear when Flx1b is inactivated (dashed lines in **B3**). These effects of Ext1b are illustrated in a functional wiring diagram (**B**, top). Effective synapses are presented with lines ending with an open triangle. Other relationships are indicated with an arrow and a question mark.

To go further in the analysis of how triphasic pattern can be elaborated in spinal sensory-motor circuits, we have examined more precisely the impact of each sensory feedback, by replaying the same example of movement as presented in Figure 9, after suppression one of the four sensory feedback. The results are presented in Figures 10, 11.

##### 3.2.3.1 Role of Flx1a

When Flx1a was inactivated (i.e., its membrane potential maintained artificially constant at −65 mV), no movement was elicited (Figure 10A1). Moreover, the membrane potential of FlxAlpha was hyperpolarized to −68 mV in the preparatory phase (see dotted red trace in Figure 10A2). Similarly, FlxPN was also hyperpolarized to −65 mV during the preparatory phase (see dotted brown trace in Figure 10A2). These results indicate that during the preparatory phase, in the normal configuration, Flx1a was continuously activating FlxAlpha (and FlxPN), but FlxAlpha did not fire because of the hyperpolarizing SET command it received. When the GO command is given at *t* = 5 s, FlxAlpha is immediately freed from this hyperpolarization and will be activated by Flx1a. Interestingly, this source of activation will be transient because as the forearm get flexed, the Flx1a signal decreases (yellow trace in Figure 10A2), eventually leading to stopping FlxAlpha and FlxPN peaks (respectively red and brown traces in Figure 10A2).

##### 3.2.3.2 Role of Ext1a

When Ext1a is deactivated (i.e., its membrane potential maintained artificially constant at −65 mV), launching the simulation leads immediately to rapid and total flexion of the forearm. (not shown). To understand what occurs, we recorded the Ext1aIN (dark-blue trace in Figure 10B1), an inhibitory interneuron (IN) that is activated by Ext1a, and that inhibits FlxAlpha. As can be observed, in normal situation, Ext1a is constantly active (around −50 mV) at initial extended arm position (dark-blue trace in Figure 10B1). This initial active state is due to the SET command sent to Ext1aIN (not shown). As a consequence, Ext1aIN continuously inhibits FlxAlpha, helping the SET command to FlxAlpha to keep this MN under threshold. When Ext1a is inactivated, this equilibrium is broken and the arm immediately starts to flex.

###### 3.2.3.2.1 Role of Ext1a on ExtAlpha peak

In normal situation, when the arm is flexing, Ext1a is activated by triceps elongation. This activity is, however, partly transitory (see light-blue trace in Figure 10B1) due to the sensitivity of spindles to speed and acceleration. The resulting Ext1a activity peak is transmitted to Ext1aIN (see dark-blue trace peak in Figure 10B1). It is also transmitted to ExtAlpha that will also depolarize (green trace in Figure 10B1). However, ExtAlpha is also activated by ExtPN (violet trace). This late activity will be added to the Ext1a activity to develop the ExtAlpha peak (vertical arrow in Figure 10B1). To help the reader, a functional diagram is presented at the top of Figure 10B1.

###### 3.2.3.2.2 Role of Ext1a on FlxAlpha peak

In normal situation, the Ext1a peak, produces a concomitant peak in Ext1aIN (see above). The activity of Ext1aIN will in turn inhibit FlxAlpha (this is the classical reciprocal inhibition circuit). Due to this inhibition, FlxAlpha will terminate its peak in advance compared to FlxPN peak (compare red and brow traces in Figure 10B2).

##### 3.2.3.3 Role of Flx1b

Keeping the same parameter set used in Figure 9, this time, we inactivated Flx1b. We still observed a movement (Figure 11A1), but it was slower (see dotted red trace, and compare to red trace). This result is in accordance with the reinforcing role of 1b afferent on their muscle during the onset of movement observed in physiology (Donelan and Pearson, 2004). Indeed, in the present simulation, in the absence of Flx1b peak there were no FlxPN and, hence, no FlxAlpha peak (see dashed brow and red traces, respectively, in Figure 11A2). See also the functional wiring diagram in Figure 11A2, top.

##### 3.2.3.4 Role of Ext1b

When ExtIb is deactivated, the flexion movement does not stop until full flexion and subsequent re-extension (Figure 11B1). In the absence of Ext1b feedback, ExtPN vanishes (violet trace in Figure 11B2), indicating that, in this example, ExtPN is most exclusively under control of Ext1b, and hence, virtually not influenced by ExtIa. From the pathway Ext1b- > ExtPN- > ExtAlphaMn reminded in the inset of Figure 11B, we can conclude that ExtPN peak suppression is responsible for ExtAlpha peak being truncated (green trace in Figure 11B2). This small reduction in ExtAlpha peak is enough to induce a total failure in the flexion stop.

More interestingly, Ext1b seems to play a key role in the production of the second FlxAlpha peak (triphasic pattern), because in the absence of Ext1b the second FlxAlpha peak totally vanishes (see dashed red curve in Figure 11B3). The suppression of the 2nd FlxAlpha peak is also linked with the disappearance of the 2nd FlxPN peak (see dashed brown curve in Figure 11B3).

##### 3.2.3.5 Which Ext1b pathway is involved in movement stop and 2nd FlxAlpha peak in triphasic pattern?

**Figure 12:**
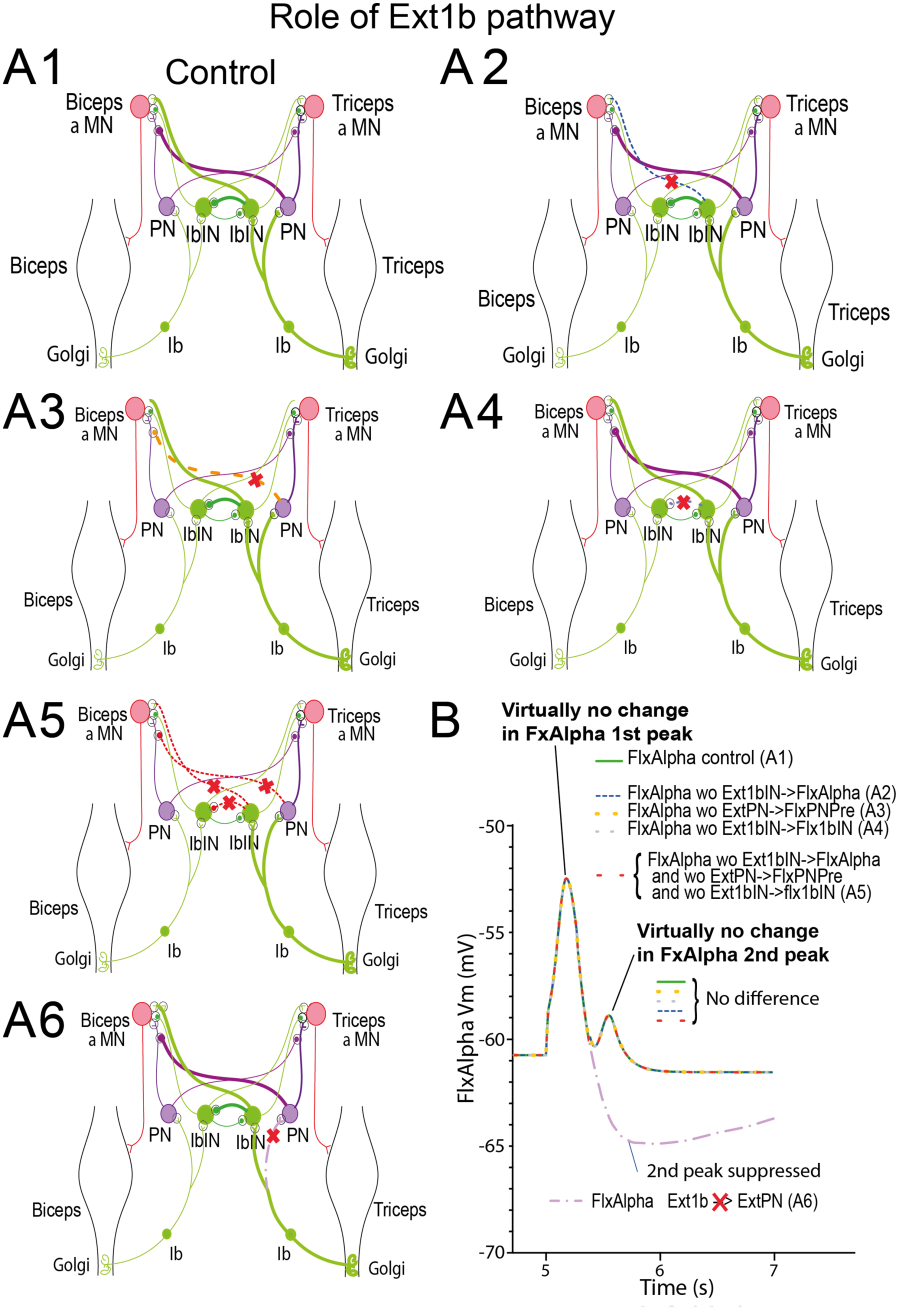
Role of Ext1b pathways in the production of the 2nd FlxAlpha peak of triphasic pattern. **(A)** Exploration of the various pathways from Ext1b to FlxAlpha **(A1)**. Several replays of the simulation of the same movement as in Figures 10, 12 have been performed in which one of the involved synapses has been suppressed in each run **(A2–A5)**. In **A6** the three synapses involved in crossing pathways have been suppressed at once. The result of the run of the model with these diverse ablations are presented in **(B)** using the same color as the suppressed synapse (see text for explanations).

From the result presented in Figure 11B3, we conclude that Ext1b is involved in the FlxAlpha 2nd peak of triphasic pattern (see functional wiring diagram in top of Figure 11B). Hereafter, we dissect out all pathways between Ext1b and FlxAlpha (Figure 12). If the effect of Ext1b onto ExtPN and ExtAlpha was demonstrated to involve effective synapses, the pathway involving Ext1bIN to FlxAlpha synapse remains to be analyzed. Moreover, the effect of Ext1b onto FlxPN is not supported by any synaptic pathway.

If we consider all these pathways (Figure 12A), it is easy to replay the simulation of this movement, after selectively suppressing a single synapse to estimate its role in the transmission of Ext1b feedback. When the Ext1bIN- > FlxAlpha synapse was suppressed (blue dashed curve in Figure 12A2) the FlxAlpha second peak was virtually unchanged (see blue dashed curve in Figure 12B). The same absence of effect was also observed when ExtPN- > FlxPNPre was suppressed (orange dashed line in Figure 12A3), or when the Ext1bIN- > Flx1bIN synapse was suppressed (orange dashed line in Figures 12A4,B) or when these three synapses were suppressed at the same time (red dashed curves in Figures 12A5, 13B). These results indicate that the second FlxAlpha peak is not due to a cross connection from Ext1b to FlxAlpha.

**Figure 13:**
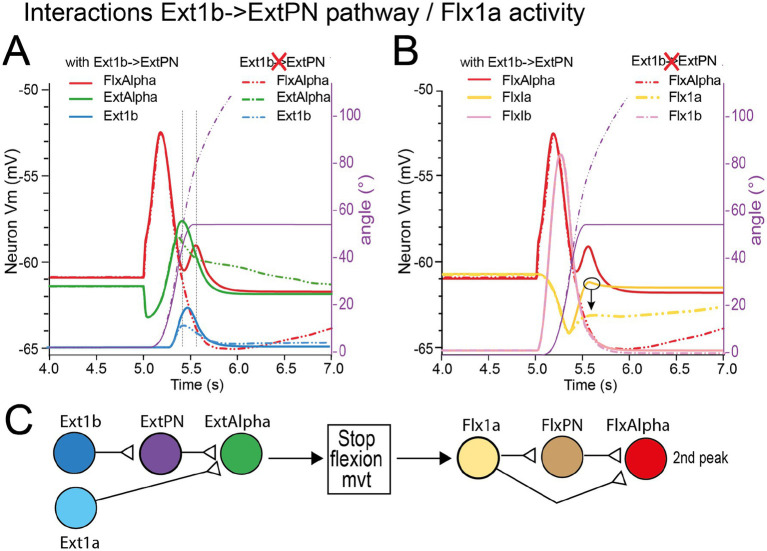
Interaction of Ext1b pathways and Flx1a activity. **(A)** Contribution of Ext1b- > ExtPN synapse on ExtAlpha activity and Ext1b feedback. **(B)** Contribution of Ext1b- > ExtPN synapse on Flx1a and Flx1b feedback. **(C)** Contribution of Ext1b- > ExtPN synapse on FlxPN activity. The FlxAlpha activity (red traces) and the flexion movement (violet traces) are presented in the two graphs **(A,B)** to indicate the timing of the second peak relative to movement.

By contrast, when the Ext1b- > ExtPN synapse was suppressed (dashed violet curve in Figure 12A6), the second FlxAlpha peak totally disappeared (dashed violet curve in Figure 12B). Consequently, if the second FlxAlpha peak is contingent upon Ext1b feedback, it is mediated not through central pathways but through a mechanical mechanism involving the reinforcement of ExtAlpha via the Ext1b → ExtPN → ExtAlpha pathway.

We tested this hypothesis in Figure 13 by suppressing the Ext1b → ExtPN synapse and assessing its effects on FlxAlpha (red), ExtAlpha (green), and Ext1b (blue) activity. Suppressing this synapse abolished the second FlxAlpha peak, modified the ExtAlpha activity profile, and reduced the Ext1b peak amplitude. Ext1b activity reinforces the ExtAlpha peak through a positive feedback loop; strengthening this feedback further elevates Ext1b activity (compare solid and dashed blue traces in Figure 13A). This feedback mechanism enhances the braking action of ExtAlpha on flexion, leading to a more rapid termination of the movement.

The rapid change in movement speed is reflected in Flx1a activity, as shown in Figure 13B (yellow curves). In the absence of the Ext1b → ExtPN synapse, the diminished braking effect leads to a slower deceleration of movement (violet trace in Figure 13B) and reduced Flx1a activity during the later phase (after *t* = 5.3 s; yellow dashed curve). Crucially, the second Flx1a peak is abolished (circled and indicated by an arrow in Figure 13B). This sensory second peak in Flx1a normally contributes to the generation of the second FlxAlpha peak through both a direct connection and an indirect pathway via FlxPN (see the second FlxPN peak, brown trace in Figure 11B3). This conclusion is summarized in the diagram presented in Figure 13C.

##### 3.2.3.6 Generalization of the mechanisms underlying triphasic elaboration process in spinal circuits

**Figure 14:**
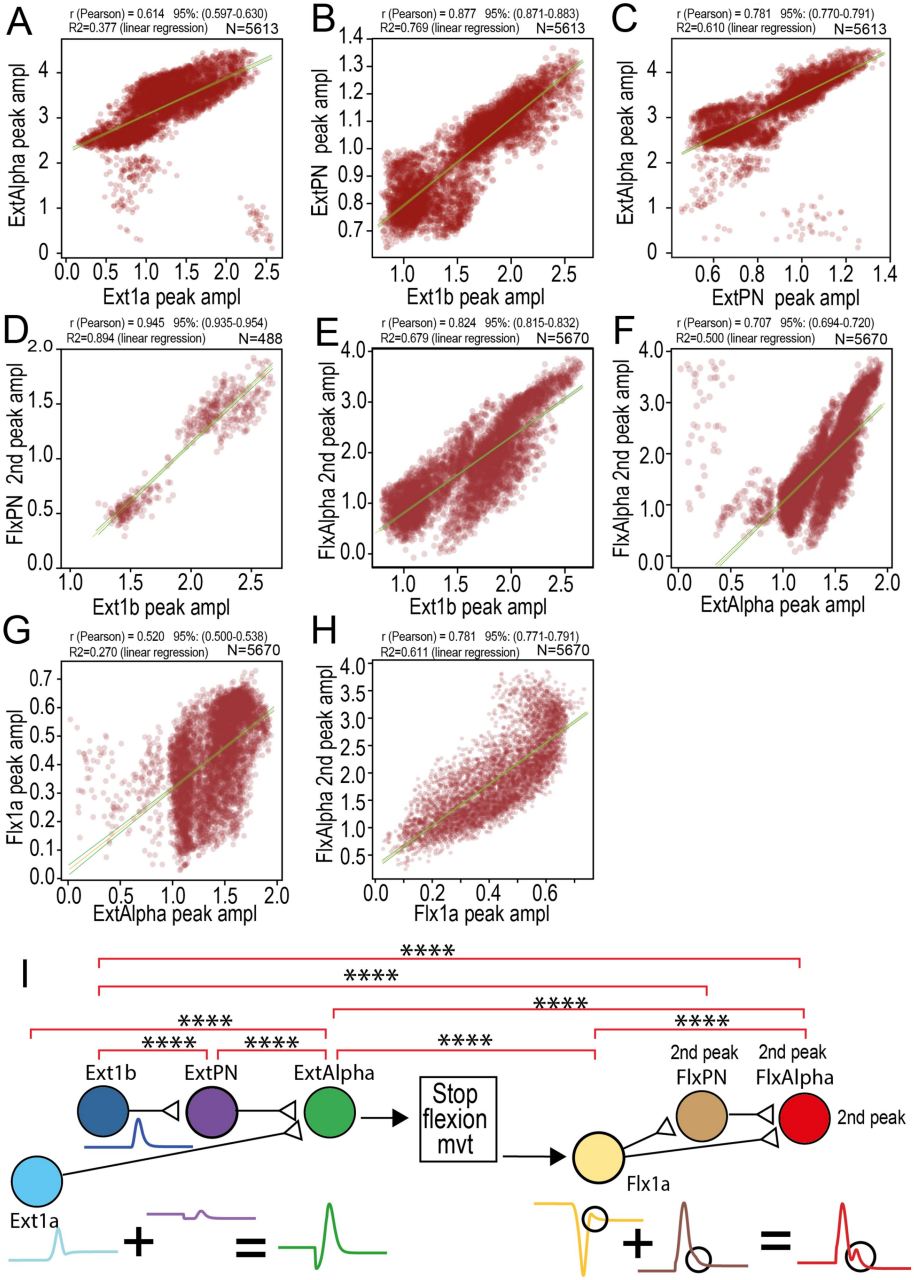
Analysis of triphasic patterns: generalization of mechanisms. **(A–H)** Correlations between the amplitudes of activity peak of all neurons implied in the occurrence of FlxAlpha 2nd peak. The analysis was made on all 5,670 valid movements presenting a triphasic pattern in the MNs (see Figure 4A). **(I)** Diagram presenting the implied neurons (with a sample of their activity leading to a triphasic pattern). Synaptic connections are represented by a line ending with an open triangle. In the middle of the diagram, the connection is not supported by a synapse but is mechanical due to abrupt flexion stop sensed by Flx1a muscle spindle, that produces a peak of activity (indicated bay a circle on the yellow trace). FlxPN 2nd peak is sometimes presented as a simple bump occurring at the end of the first peak (see circle on the brown trace).

The mechanisms leading to triphasic pattern were demonstrated for a single example of movement (see above). To evaluate how general were the relationships established in this study on the 50-parameters model, we then tested these links by correlation analysis made on peak activities of the neurons involved in the elaboration of triphasic commands recorded in Alpha MNs (Figure 14). Indeed, on the 6,706 valid movements, 5,670 displayed a triphasic pattern in Alpha MNs on which only 2032 where over threshold and produced a triphasic pattern in EMGs. Therefore, instead of analyzing only the 2032 movements associated with a triphasic EMG pattern, we performed correlation analysis on the 5,670 valid movements in which the triphasic pattern is present in the Alpha MNs.

All the links were thereby validated by significant Pearson correlation coefficients (r). As expected, ExtAlpha peak is correlated with Ext1a peak (Figure 14A, *R* = 0.614, *p* < 0.00001), Ext1b peak is linked to ExtPN peak (Figure 14B, *r* = 0.877, *p* < 0.00001), which is itself linked to the ExtAlpha peak (Figure 14C, *r* = 0.781, *p* < 0.00001). As simulated ablations demonstrated the link between Ext1b activity and the Flexor 2nd peak (see Figures 11, 12), we tested also this link with correlation analysis. Ext1b peaks were correlated with FlxPN 2nd Peak (Figure 14D, *r* = 0.945, *p* < 0.00001) and FlxAlpha 2nd peak (Figure 14E, *r* = 0.824, *p* < 0.00001). Since we concluded that the link between Ext1b and FlxAlpha 2nd peak did not involve a neural pathway, but was due to mechanical events (brutal stop of the flexion movement sensed by Flx1a – Figure 13C), we tested the correlation between ExtAlpha (responsible for this stop) and the amplitude of FlxAlpha 2nd peak. As expected ExtAlpha peak was correlated both to FlxAlpha 2nd peak (Figure 14F, *r* = 0.707, *p* < 0.00001) and to Flx1a peak (Figure 14G, *r* = 0.520, *p* < 0.00001) that is itself correlated to FlxAlpha 2nd peak (Figure 14H, *r* = 0.781, *p* < 0.00001). The corresponding R2 values are all indicated above each correlation graph, together with the confidence intervals of r Pearson correlation coefficients. Confidence intervals of the correlation (95%) is indicated by two green curves on each side of the orange correlation line.

## 4 Discussion

In the present modelling work, we explored the capacities of spinal sensorimotor networks to produce triphasic EMG pattern when activated by simple SET and GO step commands. We observed spontaneously occurring triphasic pattern EMGs produced by spinal sensorimotor circuits (50-parameters model) during the goal exploration process used to establish the behavioral capabilities of the system. Critically, the timing of such triphasic patterns were not predetermined in the simple step functions controlling their state. Particularly, the GO command, initiated the movement (*t* = 5 s) but did not give any indication for the observed triphasic EMG patterns. It is important to note that such triphasic pattern were observed in a large range of rapid movements in the behavior domain (Figure 8B). Therefore, triphasic pattern production for any movement seems to be an intrinsic property of sensorimotor spinal network in interaction with musculoskeletal system via MN activities and proprioceptive feedback. This finding is significant as it reveals the spinal circuit’s ability to generate complex muscle activity, such as the triphasic pattern, by relying on proprioceptive feedback loops, as previously hypothesized (Barnett and Harding, 1955; Terzuolo et al., 1974; Ghez and Martin, 1982; Feldman, 1986, 2019). In this study, we modeled spinal circuits and examined how they process proprioceptive feedback to generate triphasic commands, given that network properties were finely tuned by descending step commands (SET and GO).

The role of proprioceptive feedback in shaping triphasic commands within spinal circuits was previously proposed by Feldman and colleagues (Feldman, 1986, 2019). However, our work differs from the lambda model theory in that our model does not incorporate equilibrium point control. Interestingly, similar to the lambda model, our model demonstrates that non-patterned commands (step commands) can give rise to patterned agonist–antagonist muscle activity (Gribble et al., 1998). This convergence between the two models is noteworthy because, despite the absence of a global control parameter such as the equilibrium point in our model, future research could explore how the valuable insights of the lambda model might emerge from a part of the solutions uncovered by Goal Exploration Process (GEP). Additionally, our study highlights the diversity of valid descending command sets (SET and GO) identified through GEP. This diversity arises from the redundancy of parallel sensory-motor pathways within the spinal cord—an aspect not addressed in the lambda model.

Since the 50-parameters model involved both Ia spindle and GTO afferent feedback, we have used a dissecting out approach to evaluate the role of Ia and Ib afferents and their respective sensorimotor circuits in the elaboration of the triphasic commands.

### 4.1 Necessity of spindle and GTO feedbacks in triphasic pattern

Removing Ia sensory feedback totally prevented the occurrence of any valid movement (Figure 7B), indicating that the inherent capacity of spinal sensorimotor circuits to elaborate valid movements when interacting with a musculoskeletal system, requires spindle afferent signals.

By contrast, removing Ib sensory feedback and Ib sensorimotor circuits did not prevent spinal sensorimotor circuits to produce minimum jerk movements (Figure 5B). However, in the absence of GTO feedback, the extend of the behavior domain was greatly reduced (compare Figures 4B, 5B). More strikingly, the absence of GTO feedback totally prevented the occurrence of triphasic commands, and indicates that although Ia feedback and Ia sensorimotor spinal circuits can handle the elaboration of minimum jerk movements, Ib afferent signals and Ib sensorimotor circuits are necessary (in addition to 1a sensory-motor circuits) to produce triphasic commands in our model. Among reciprocal connections from Extensor sensory feedback (i.e., ExtIaINs and IExtbIN) only Ext1aIN could be involved in the production of triphasic pattern, specially by terminating the first FlxAlpha peak more rapidly (Figure 10B). ExtIbIN seems not necessary for triphasic pattern because when its cross connections are suppressed, the second Flexor peak is unaffected (Figure 12A5). Therefore, this study demonstrates that spinal sensorimotor networks fed by spindle afferent signals and GTO afferent signals, have the capacity to handle the production of triphasic command pattern in a large range of fast and large movements of the behavior space.

It is then highly likely that spindle and GTO afferents participate in the elaboration of this triphasic command in animals and human. But if this conclusion is true, how to explain the finding that triphasic patterns were still produced in transient deafferentation experiments (Sanes and Jennings, 1984)? In fact, this experiment does not totally invalidate the participation of sensorimotor circuits in the shaping of the triphasic command, because the ischemic block affected the afferent activity of the muscles below the elbow, leaving intact both the efferent copy of their motoneurons via Renshaw’s interneurons and all proprioceptive feedbacks from all proximal muscles. The widespread extent of these feedback circuits (Cavallari et al., 1992), and the strong modulation of proximal muscles to stabilize posture during distal phasic movements, were not fully appreciated until later (Massion, 1992).

### 4.2 Role of spindle feedback in triphasic pattern

We have shown that 1a feedback is essential for the spinal sensorimotor circuits to handle valid movements. Indeed, it is interesting to address the question of how simple step commands can elicit dynamic responses in motor activities, i.e., transient activation of flexor at two times (i) for launching the movement, (ii) just before stabilization at the end of the movement, and transient activation in the extensor to stop the movement.

Let us start with the first Flx transient activation. When we look at the result of suppressing Flx1a feedback (Figure 10A), we had noted that without Flx1a feedback the movement did not start, because the strategy uncovered by GEP consisted in a strong enhancement of Flx1a sensitivity to maximize its discharge during the preparatory phase. To avoid this sensory activity to activate FlxAlpha MN, the SET command sent a hyperpolarizing order to FlxAlpha MN. When the GO command was sent to FlxAlpha MN, this hyperpolarizing action was suppressed and the Flx1a sensory feedback could strongly activate FlxAlpha MN. But, because movement was launched, the biceps muscle started to shorten and Flx1a signal decreased (see yellow curve in Figure 10A). This is how the step command elicited a transient activation of FlxAlpha MN, via sensory coding of muscle length (i.e., via the movement itself).

The transient activation of ExtAlpha MN (Figure 9E, green curve) is also directly related to Ext1a feedback (Figure 9E, blue curve). But its transient nature is related to the dynamic response of Ext1a stretch (i.e., to the speed of the stretch – see Figure 3A). Note that a possible improvement of our model of spinal sensorimotor circuit would be to introduce both dynamic and static gamma commands. This would improve handling of 1a feedback transients. This was not possible in AnimatLab but future work should address this question.

### 4.3 Role of GTO feedback in triphasic pattern

It seems that 1b feedback is necessary for occurrence of triphasic command, but not sufficient because it cannot produce triphasic command without spindle feedback. So, how 1b feedback can help 1a afferent feedback for triphasic pattern production in spinal sensorimotor circuits? If we look at the activity of FlxPN (Figure 9D) and Flx1b (Figure 9F) in the 50 parameters model, we note that they display similar activity along time course. This indicates that Flx1b signals play a great role in assisting the Flx activity at movement onset. When Flx1b feedback is suppressed, maximal speed is reduced (see Figure 11A1). More importantly, the transient FlxAlpha peak totally disappears (compare red dashed line and red curve in Figure 11A2). This means that Flx1b and Flx1a activities are both necessary to launch the movement. Then while the muscle shortens, Flx1a activity decreases rapidly, and because the force produced by the biceps muscle decreases due to (i) muscle shortening and (ii) to FlxAlpha MN activity decrease (consecutive to Flx1a decrease), the activity of Flx1b becomes transient. This involvement in force enhancement was also described in physiological conditions in cat (Donelan and Pearson, 2004). It seems that 1b contribution consists mainly in this enhancement in the flexor phase of the movement (i.e., movement initiation), but 1b feedback seems also involved in reinforcing ExtAlpha MN activity when the extensor is stretched during the flexion movement (compare ExtAlpha activity with and without Ext1b feedback in Figure 11B) and certainly contributes to shortening the brake phase. Therefore, in the absence of 1b feedback, the brake is less powerful and this phase is too slow for a minimum jerk profile (compare end of movements in the complete model – Figure 4C1, with the model derived of 1b feedback – black arrow in Figure 5C1). Ext1b peak induces a rapid slowdown at the end of the movement, which is responsible for the peak of Flx1a activity (due to velocity and acceleration sensitivity of 1a afferents - see yellow trace in Figure 13B). Consequently, this Flx1a peak, in turn activates the Flx alpha MN (red trace in Figure 13B). In addition, Flx1b also sense this rapid brake and may produce a second peak (mostly if an overshot is present on the movement – data not shown). Finally, both Flx1a peak and FlxPN 2nd peak converge onto FlxAlpha MN (see yellow, brown and red trace in Figure 14I).

The present findings align with physiological studies highlighting the role of 1b positive feedback in muscle activity during motor control (Donelan and Pearson, 2004). However, since our model operates in the absence of gravity, the additional force generated by GTO positive feedback counteracts limb inertia rather than gravity. Consequently, without gravitational influence, no force was needed to maintain a position, and the GTO contribution remained purely dynamic, with no static component. Due to this limitation and the simplicity of the present model (a single degree of freedom movement with only two antagonistic muscles), we were unable to investigate how GEP would have identified solutions in which both excitatory and inhibitory components contribute to interjoint coordination in complex movements (Nichols, 2018). It will be interesting, in future work, to explore this possibility.

### 4.4 Role of descending commands and spinal networks in triphasic pattern

The fact that Ia spindle and Ib feedbacks and their spinal sensorimotor circuits are capable of handling triphasic commands all over the behavior domain, does not exclude, however, that in physiological conditions, descending command also participate in the elaboration of triphasic commands. Recordings from M1 motor cortex indicate that unit and global activities can be correlated either with static and dynamic forces and torques around single joints (Cabel et al., 2001), or with muscle synergies (Holdefer and Miller, 2002). However, the question of the origin of triphasic activities in M1 is still open because correlation is not causation, and triphasic activities in M1 could simply result from efference copy from spinal interneurons and afferent feedback from proprioceptors. Whatever the origin of M1 triphasic activity, it is likely that such precise descending command interact with spinal sensorimotor networks. Future research in this domain will have to unravel how complex descending commands interact with spinal sensorimotor process that can handle by itself triphasic commands.

In the present report, we used simple step commands to control spinal sensorimotor circuits. Similarly, some authors have argued that because EMGs are organized in triphasic pattern with silent periods, this indicated that descending commands did not operate in a smooth and continuous manner (Leib et al., 2020). The bang–bang theory is a control model that predicts the triphasic pattern of muscle activity observed during hand reaching. In this framework, the motor plan for muscle activation is converted into a piecewise-constant control signal, which is then low-pass filtered and transmitted to the muscles with muscle-specific delays, estimated using a Markov chain Monte Carlo (MCMC) approach. This bang–bang controller suggests that the brain reduces the complexity of ballistic movement control by issuing motor commands at sparse, discrete time points. Indeed, the simple model (bang-bang optimal control model) proposed by these authors is more structured in time than the simple step functions we used here. In the bang-bang control, the control signal abruptly switches between a maximum and a minimum value at certain sparse points in time. The control function is made of three constant segments (with two transitions). The bang-bang model explains both abrupt changes in muscles activity (triphasic command) and the smooth reaching trajectory. Interestingly, rGEP was capable of uncovering a set of valid movements (minimum jerk movements) relying incidentally on triphasic commands (with abrupt changes in activity), although this triphasic command was not explicitly searched for nor included in the process with simple step commands. Therefore, it is possible that spinal network properties (and the associated biomechanical effector system) possess the capacities of handling such complex dynamic commands just by giving the correct level of stimulation of target spinal neurons and precise synaptic gain settings. In a more general perspective, these spinal sensorimotor circuits could be considered as the physiological support of movement primitives proposed by Degallier and Ijspeert (2010) that can be handled by simple step commands from the brain.

In this case, the motor primitives not only encompass movement’ start dynamics but also movement’ stop dynamics with a unique step command. Notably, the precise timing of ANT and AG2 bursts is essential for the triphasic pattern to produce smooth, minimum-jerk movements. Other models using artificial neural networks to generate internal representations for controlling ballistic movements via step functions have highlighted the critical role of precise step function timing in achieving such movements (Bernabucci et al., 2007). Interestingly, in our model, although the timing of step functions is fixed, the precision of motoneuronal bursts is entirely regulated by spinal sensorimotor networks and the musculoskeletal system. This is particularly noteworthy because the argument for precise timing has often been used to support the necessity of a central origin for the triphasic command. However, our findings demonstrate that such precision can emerge from spinal sensorimotor mechanisms.

### 4.5 Triphasic pattern and minimum jerk

Simulation studies have suggested that minimum-jerk models (Flash and Hogan, 1985) and minimum torque-change models (Uno et al., 1989) were likely not capable to predict muscle actions (Osu et al., 2004) and thus triphasic pattern. Here, we used a different approach that involved a musculoskeletal model associated to spinal sensorimotor networks, and a search method (rGEP) to revisit the capability of spinal sensorimotor circuits to produce triphasic pattern of muscle activation.

Indeed, rGEP uncovered a great diversity of parameters associated with a given valid movement. Among these parameters, some will result in triphasic commands. This solution was found all over the behavior domain of the model. But other parameter sets do not fit the definition of triphasic command. For example, although the silence of the agonist between the two agonist peaks (AG1 and AG2) was found in most of the 50-parameters model valid movements (Figure 9B), there were also solutions with no 2nd Flx peak all over the behavior domain of this complete model.

The fact that we imposed a minimum jerk profile for movements had consequences on the type of triphasic command retained by rGEP. Indeed, when we consider fast movements that were retained to define the triphasic pattern (Hallett et al., 1975), a small oscillation at the end of the movement is often present. The origin of this final oscillation comes from ANT peak that was slightly too strong, resulting in a final oscillation that required the second agonist peak (AG2) to be damped (Berardelli et al., 1996). The minimum jerk profile virtually imposes the absence of such oscillation at the end of the movement, and thereby reduces dramatically the amplitude of AG2.

### 4.6 Comparison with other models

These results can be compared to those obtained from the mathematical model of the arm musculoskeletal system proposed by Houk and Barto (2000). In that model, the lambda (*λ*) approach generated bell-shaped velocity profiles (i.e., minimum jerk movements) by directly shifting the equilibrium point to the desired target. This model leverages the damping properties of muscles produced by the stretch reflex.

However, it is important to note that in Houk’s model, step functions do not represent instantaneous jumps from one λ value to another. Instead, they involve gradual, time-dependent changes in motor commands (λm). A second key difference is that a simple step-function change in the equilibrium point was insufficient to produce true minimum jerk movements; instead, a pulse-step adjustment was required. A third distinction is the presence of coactivation before and after movement.

Despite these differences, as mentioned earlier in the discussion, the convergence between the lambda model and our model highlights the need for further investigation into the origins of these similarities—particularly in exploring the space of descending commands (i.e., the parameter space).

A more comprehensive version of the lambda model, known as the “referent control model” (for an overview, see Feldman, 2015), suggests that by modifying the rate and duration of shifts in λ thresholds, the system can influence movement speed and amplitude. More broadly, these shifts can also explain rapid transitions between two states where EMG activity is entirely absent, yet the system remains close to activation (i.e., the threshold position is reset between the initial and final positions) (Ostry and Feldman, 2003; Feldman, 2015). The referent control model also accounts for dynamic control via velocity-coded Ia feedback. Given these considerations, the lambda model appears to share common output characteristics with our model. Both models adhere to the physical principles that govern force generation in muscles, which depend on muscle properties and activation by motoneurons, controlled through proprioceptive feedback (either directly or via propriospinal interneurons) and descending commands acting on propriospinal interneurons.

However, our model differs in that it does not rely on threshold shifts. Instead, it explores all possible motor control mechanisms present in spinal cord circuits, which integrate proprioceptive feedback (from muscle spindles and Golgi tendon organs) and descending commands onto spinal interneurons. To investigate these spinal control mechanisms, we also employed step functions, but their nature is fundamentally different from those used in Houk and Barto (2000). In our model, step functions regulate neuron excitability and synaptic strength during the preparatory phase (SET), and only eight step functions acting on spinal interneurons are used to initiate movement (GO).

While our step commands are certainly open to debate—since descending commands are likely far more complex—our approach has the advantage of producing movements based on spinal sensorimotor circuit control, where the time course of activity of each component remains accessible. In future work, it would be interesting to use our model to test hypotheses proposed in the referent control model. Because we have access to the state of all neurons and muscles at any given time, we could analyze which parameter sets, as uncovered by GEP, align with a simple λ threshold shift and which do not—while still leading to valid movement.

### 4.7 Interest of rGEP compared to CMAes in the search for behavior domain

CMAes is an optimization algorithm. It is useful to find a set of parameters that will produce a specific target movement. However, the use of CMAes to uncover the totality of the behavior domain is not adapted. This procedure generally requires 500 runs to eventually find a valid behavior if convergence occurs. This means that some targets will not be found by CMAes, although they exist. The reason for these failures comes from the multiplicity of local minima (mentioned in the introduction), and to the diversity of parameter sets that exist for a given movement (redundancy problem). Moreover, the use of CMAes to uncover the behavior domain would require to define a great number of targets in the behavior space to find the borders of the domain. Such a search, in addition to the high rate of failures, would have required far too many runs with no certainty of positive result. At the limit, we could have imagined a mixt of GEP and CMAes in which new targets are searched in the proximity of a pre-existing one (like the extend process), by replacing the random modification of target’s parameters by a CMAes pointing directly on searched movements around the target. However, this mixt approach would have multiplied the computer time by at least by 100 times. Instead, rGEP progresses from known behaviors to discover new ones by single trials with no optimization. GEP algorithms are therefore very effective (sample efficient) as stated in the introduction.

### 4.8 Limits and perspectives of the study

#### 4.8.1 No separate static and dynamic gamma MNs

All models of the present study used a mixed type of gamma MN (one for the biceps and one for the triceps). Ideally, we should have used two types of gamma MNs for each muscle: a static gamma MN and a dynamic gamma MN, as was the case in the model of spinal-like regulator (Raphael et al., 2010), but these distinct gamma control does not exist at present in AnimatLab. However, the activation of the gamma MN of our model increased spindle sensitivity to both position and velocity, and this single type of gamma MN was enough to produce the desired behavior of the model under rGEP. Certainly, the possibility for the step commands to act differently on spindle length coding and speed/acceleration coding feedbacks should benefit to the possibilities of control (see “Role of spindle feedback in triphasic pattern” above), and likely extend the behavior domain of the model. This point will need future work to estimate the changes brought by the two gamma MN types.

#### 4.8.2 Absence of gravity

We presented here the results of movements conducted in the absence of gravity, equivalent to the elbow movement studied in Hallett et al. (1975). However, the presence of gravity during elbow flexion would exert an increasing torque that opposes the action of the biceps and could potentially obscure the role of the triceps in slowing down the movement, particularly when the movements are not sufficiently fast.

It will be interesting in future investigations to add gravity to see how triphasic pattern evolve, depending on movement trajectory, and how spinal network can handle it. Such studies should show smaller ANT burst when gravity is present and tend to slow down the movement as the forearm becomes more horizontal during flexion movements. Inversely, if movements start from a flexed position (say 110° in our model), and stops at 20°, ANT burst should be dramatically increased and thus AG2 burst should increase accordingly. Therefore, in both conditions, our model would remain valid (as confirmed by preliminary simulations - data not shown).

#### 4.8.3 Simple flexion movement

In the present study, we used a single joint (elbow) and movement consisted in elbow flexion. This was made because minimum jerk is more adapted to this situation (Nakano et al., 1999). It would be interesting to generalize the results of the present study by using more complex models including two joints, and mono- and bi-articular muscles, and considering endpoint reaching movements as was used in Tsianos et al. (2014). In this view, it would be interesting to test the roles of GTO feedback: excitatory force feedback contributing to propulsion and weight-bearing tasks, while inhibitory force feedback contributing to inter-joint coordination (Nichols, 2018). Moreover, such models would make it possible to test complex interactions between GTO and muscle spindle feedback, and to analyze how their combination helps manage inter-joint coordination and stabilize limb mechanics against inertial forces during movements (Nichols, 2018).

#### 4.8.4 Absence of co-contraction in preparatory and post-movement phases

In the present work, we have chosen not to allow co-contraction during the preparatory phase and after movement was achieved and position was maintained. Indeed, in similar experiments, using elbow flexion on a horizontal surface with reduced friction (Hallett et al., 1975), in which case gravity does not exert any role, a slight residual tonic activity is often observed after movements was achieved. This residual tonic activity was also observed in the simulations of Raphael et al. (2010), using a similar model as the one we used in our study. As mentioned above, such residual activity was observed if the cost function did not penalize them. Indeed, a whole range of activity level was then observed post-movement. But it was accompanied with a corresponding degree of co-contraction in antagonistic muscles. In human, co-contraction is also favored when movements are made in a perturbing environment (Milner and Cloutier, 1993). By increasing stiffness, the co-contraction provides a good way to resist perturbations or at least to diminish their effects. An extension of the present work could be to vary the allowed degree of co-contraction in the post-movement phase and analyze the effects of perturbations on the resulting behavior map and on the valid solutions it contains.

#### 4.8.5 GEP and exploration of redundancy

The systematic investigation of the behavioral constraints of reduced spinal circuitry highlights the functionality of a system that might otherwise be considered excessively redundant. For organisms navigating real-world environments, it may be more important to get the largest possible range of performances from their musculoskeletal systems rather than concentrating on the narrow range of tasks typically examined in laboratory settings. In this line of thought, a potential development of the present work would be to evaluate the adaptative role of redundancy by introducing unexpected perturbations or constraints, and see how redundancy helps the system to find appropriate solutions. Notably, such research would highlight the importance of spinal networks in facilitating rapid adaptation under the brain’s supervision as is the case in reservoir computing (Tanaka et al., 2019).

#### 4.8.6 Multiplicity of descending commands

In the present model, we used simple step commands to demonstrate the intrinsic ability of spinal sensorimotor networks to manage the complex dynamics of a triphasic pattern. The simplicity of these step commands is offset by their multiplicity—50 descending step commands. However, this apparent complexity does not imply that all 50 parameters need to be adjusted for each movement. In fact, while some parameters must be precisely tuned, others have little to no impact on movement execution. Future research could explore which parameters are crucial for determining movement amplitude and speed. This question is closely related to the issue of redundancy, as multiple parameter configurations can produce the same movement. Additionally, it is possible that certain predefined SET commands could position the network in a configuration beneficial for all movements, while only a few specific commands determine the characteristics of each movement.

Finally, the present modelling study offers an opportunity to test the physiological roles of Ia and Ib proprioceptive feedback in voluntary movements. Since the model pointed out the specific roles of Ia and Ib at specific timings, it should be possible to test if the involved circuits are responsible for the elaboration of the triphasic commands observed during rapid arm flexion, for example by delivering perturbations at these specific time windows and see if muscle responses are in accordance with similar perturbations delivered in the model.

## Data Availability

Python scripts for GEP and all simulations included in this study can be downloaded from GitHub, with an installation procedure for all software package involved: (https://github.com/Cattaert/rGEP/tree/main).
