# Supplementary material for: Role of spinal sensorimotor circuits in triphasic muscle command: a simulation approach using goal exploration process
Source: Front Comput Neurosci. 2026 Mar 11;20:1745836. doi: 10.3389/fncom.2026.1745836 (PMC13015193; doi:10.3389/fncom.2026.1745836)
Supplement: Supplementary file 1 [file Table_1.docx]

**Supplementary materials**

- 1. *Pseuco code illustrating rGEP algorithm*

**Input**: *original seeds* $\left\{ \left( x_{j},y_{j} \right) \right\}_{1\leq j\leq n_{S}}\in(B_{V}\times P_{V})$

**Input**: *Sigma*

**Input**: (*Nb_Expand, Nb_Fill)*

**Output**:
$H_{B} = \left\{ \left( x_{j},y_{j} \right) \right\}_{0\leq j\leq n_{B}}\in(B_{B}\times P_{B})$ avec

$$H_{B}\leftarrow seeds$$

$\mathbf{for} run = 0 \to\mathrm{Nb}_{\mathrm{Expand}} \boldsymbol{do}$

*B_t_* = $getExpTargets(H_{B})$

(*B_v_,P_v_)* = $getClosest\_Valid\_Behavs(B_{t})$

*P* = $randChangeParam(P_{v})$

*NB* = $MakeSimul(P)$

$(NB_{v}, NP_{v})$ = *Valid*$(NB)$

$H_{B}\leftarrow(NB_{v}, NP_{v})$

$\mathbf{for} run = 0 \to\mathrm{Nb}_{\mathrm{Fill}} \boldsymbol{do}$

*B_t_* = $getFillTargets(H_{B})$

(*B_v_,P_v_)* = $getClosest\_Valid\_Behavs(B_{t})$

*P* = $randChangeParam(P_{v})$

*NB* = $MakeSimul(P)$

$(NB_{v}, NP_{v})$ = *Valid*$(NB)$

$H_{B}\leftarrow(NB_{v}, NP_{v})$

valid behaviors (B_V_) and their parameters (P_V_)

generally, *n_S_*=3

variance of the random modifications applied to parent parameter sets

Number of repetitions of “expand” and “fill” procedures

Observation history (B_B_ =behaviors; P_B_ =parameter sets)

Starts by including the seeds in observation history

Explores the borders of the behavior domain

Updates the history

Explores the holes within the behavior domain

Updates the history

With included procedures:

**Procedure** $randChangeParam(P_{v})$

According to *Sigma* generates random values that are added to the selected valid parent parameter sets.

*Returns*: parameter sets to run: *P*

**Procedure** $MakeSimul(P)$

Run the neuromechanical simulations. The number of parameter sets depends on the $getExpTargets(H_{B})$ procedure: The largest the behavior domain, the more parameter sets to run. *Returns* the new behaviors (NB)

**Procedure** *Valid*$(NB)$

For each New Behavior obtained (NB) the cost function is calculated, and, if satisfies the validity criteria, the (New valid behaviors, New parameters) sets are returned.

- 1. *PseudCode illustrating getExpTargets(H_B_)*

**Procedure** $getExpTargets(H_{B})$

*DensMap, grid*$H_{B}$*= G*$etContDensityMap(H_{B})$

*For x in grid*$H_{B}(x)$*:*

*For y in grid*$H_{B}(y)$*:*

*getBorder_lowx_y(DensMap)*

*getBorder_upx_y(DensMap)*

$B_{t}\leftarrow$*Border_lowx_y*

$B_{t}\leftarrow$*Border_upx_y*

*For y in grid*$H_{B}(y)$*:*

*For x in grid*$H_{B}(x)$*:*

*getBorder_leftfy_x(DensMap)*

*getBorder_righty_x(DensMap)*

$B_{t}\leftarrow$*Border_leftx_y*

$B_{t}\leftarrow$*Border_rightx_y*

*Returns*: list of behavior targets: $B_{t}$

Find targets in the periphery of the current H_B_

Gets the density Map and grid from normalized $H_{B}$ (*NormHB)*

Gets the (x, y) coordinates of low and up limits in *NormHB*

Gets the (x, y) coordinates of left and right limits in *NormHB*

- 1. *PseudoCode illustrating getFillTargets(H_B_)*

**Procedure** $getFillTargets(H_{B})$

*DensMap, grid*$H_{B}$*= G*$etContDensityMap(H_{B})$

*For x in grid*$H_{B}(x)$*:*

*list =* *[(x,y) for y in grid*$H_{B}\left( y \right)$ *if (DensMap(x,y)* *=0)]*

*get starts and ends of domains (consecutive 0 densities)*

$B_{t}\leftarrow$ *list[start, end]*

*For y in grid*$H_{B}(y)$*:*

*list =* *[(x,y) for x in grid*$H_{B}\left( x \right)$ *if (DensMap(x,y)* *=0)]*

*get starts and ends of domains (consecutive 0*

*densities)*

$B_{t}\leftarrow$ *list[start, end]*

*Returns*: list of behavior targets: $B_{t}$

Find targets in holes present in the current H_B_

Gets the density Map and grid from normalized $H_{B}$ (*NormHB)*

Gets the (x, y) coordinates of low and up limits of each hole in *NormHB*

Gets the (x, y) coordinates of left and right limits of each hole in *NormHB*

- 1. *Normalization of params*

|  | Min value | Max value | Norm min | Norm max |
| --- | --- | --- | --- | --- |
| Current injection | -20 nA | +20 nA | 0 | 1 |
| Synaptic Strength | 0 | 50 uS | 0 | 1 |

if $V_{Pre}\geq V_{Thr}$

$I_{syn}=gMax\times(V_{Pre}-V_{Thr})\times\left( V_{Post}-V_{Eq} \right)$

else

$I_{syn}=$ 0

$I_{syn}$: synaptic current; *gMax*: Maximal value of synaptic conductance (S); $V_{Pre}$:Membrane potential of presynaptic neuron; $V_{Thr}$: presynaptic activation threshold (= -65mV); $V_{Post}$: post-synaptic membrane potential; $V_{Eq}$: Equilibrium potential of the non-spiking synapse (post-synaptic) ($V_{Eq}$ = -10 mV).
